# Mitochondrial Carriers Regulating Insulin Secretion Profiled in Human Islets upon Metabolic Stress

**DOI:** 10.3390/biom10111543

**Published:** 2020-11-12

**Authors:** Cecilia Jimenez-Sánchez, Thierry Brun, Pierre Maechler

**Affiliations:** Department of Cell Physiology and Metabolism & Faculty Diabetes Center, University of Geneva Medical Center, 1206 Geneva, Switzerland; Cecilia.Jimenez-Sanchez@unige.ch (C.J.-S.); thierry.brun@unige.ch (T.B.)

**Keywords:** pancreatic islets, β-cell, mitochondria, diabetes, glucotoxicity, glucolipotoxicity, lipotoxicity

## Abstract

Chronic exposure of β-cells to nutrient-rich metabolic stress impairs mitochondrial metabolism and its coupling to insulin secretion. We exposed isolated human islets to different metabolic stresses for 3 days: 0.4 mM oleate or 0.4 mM palmitate at physiological 5.5 mM glucose (lipotoxicity), high 25 mM glucose (glucotoxicity), and high 25 mM glucose combined with 0.4 mM oleate and/or palmitate (glucolipotoxicity). Then, we profiled the mitochondrial carriers and associated genes with RNA-Seq. Diabetogenic conditions, and in particular glucotoxicity, increased expression of several mitochondrial solute carriers in human islets, such as the malate carrier DIC, the α-ketoglutarate-malate exchanger OGC, and the glutamate carrier GC1. Glucotoxicity also induced a general upregulation of the electron transport chain machinery, while palmitate largely counteracted this effect. Expression of different components of the TOM/TIM mitochondrial protein import system was increased by glucotoxicity, whereas glucolipotoxicity strongly upregulated its receptor subunit TOM70. Expression of the mitochondrial calcium uniporter MCU was essentially preserved by metabolic stresses. However, glucotoxicity altered expression of regulatory elements of calcium influx as well as the Na^+^/Ca^2+^ exchanger NCLX, which mediates calcium efflux. Overall, the expression profile of mitochondrial carriers and associated genes was modified by the different metabolic stresses exhibiting nutrient-specific signatures.

## 1. Introduction

**Figure 1:**
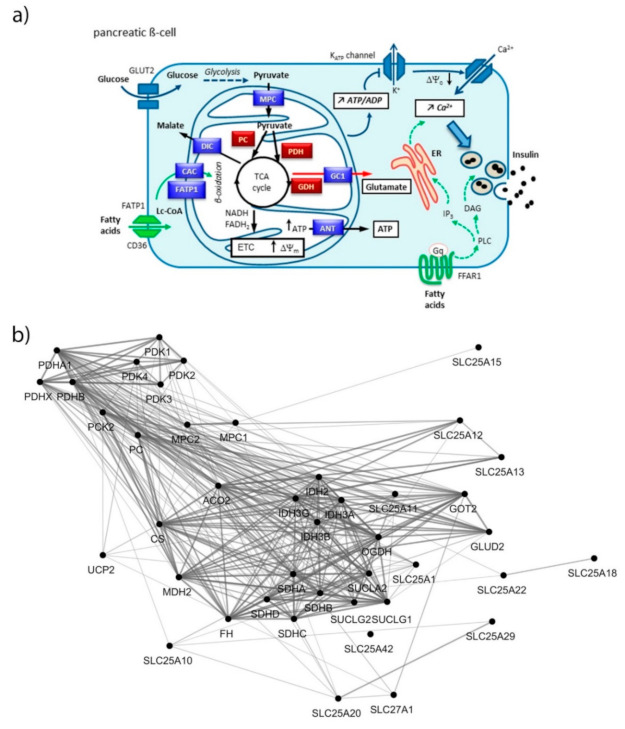
(**a**) Coupling of glucose metabolism with insulin secretion in pancreatic β-cell. Glucose is metabolized through glycolysis that produces pyruvate. Pyruvate enters into mitochondria through the mitochondrial pyruvate carrier (MPC) and fuels the TCA cycle by the action of both pyruvate carboxylase (PC) and pyruvate dehydrogenase (PDH). The TCA cycle generates reducing equivalents transferred by NADH and FADH_2_ to the electron transport chain (ETC), leading to the hyperpolarization of the mitochondrial membrane (ΔΨ_m_) and generation of ATP. Then, ANT transfers ATP to the cytosol, raising the ATP/ADP ratio that induces the closure of the K-ATP channels promoting plasma membrane depolarization (ΔΨ_c_). This opens voltage sensitive Ca^2+^ channels, increasing cytosolic Ca^2+^ concentration ([Ca^2+^]_c_), which triggers insulin exocytosis (triggering pathway, blue arrows). The amplifying pathway of metabolism–secretion coupling is contributed to by additive coupling factors, e.g., glutamate produced by glutamate dehydrogenase (GDH) and transported by GC1 (red arrow). Free fatty acids potentiate glucose-stimulated insulin secretion through long-chain acyl-coenzyme A (Lc-CoA), glycerolipid/free fatty acid cycle (not shown), import into mitochondria through the fatty acid transport protein (FATP1) and the carnitine/acylcarnitine carrier (CAC), β-oxidation; or through FFAR1 signaling (green arrows). Gαq activates phospholipase C (PLC) producing both inositol trisphosphate (IP3), which triggers calcium release from the endoplasmic reticulum (ER) stores, and raises diacylglycerol (DAG), an activator of the phosphokinase C (PKC) involved in insulin exocytosis. (**b**) Functional interaction network of mitochondrial metabolite carriers and associated genes, i.e., pyruvate metabolism, TCA cycle, amino acid metabolism and fatty acid transport. Nodes were connected using the STRING interaction knowledgebase with a confidence score > 0.4.

Pancreatic β-cells secrete insulin in response to the elevation of circulating glucose, thereby maintaining euglycemia. Once in the β-cell, glucose is processed through glycolysis and the thus formed pyruvate enters the mitochondria where it is transformed into intracellular signals leading to the stimulation of insulin exocytosis. This metabolism–secretion coupling requires efficient mitochondrial metabolism, primarily implicating pyruvate import and ATP production. The latter induces cell membrane depolarization that promotes a rise in cytosolic Ca^2+^ concentration (Figure 1). Elevation of cytosolic Ca^2+^ is rapidly transferred into mitochondria, which results in further activation of mitochondrial metabolism. While cytosolic Ca^2+^ is an obligatory signal, this ion is not sufficient to promote the full secretory response. Accordingly, metabolism in general and mitochondria in particular produce additive factors, which participate in the amplifying pathway of the Ca^2+^ signal [1]. Collectively, these mechanisms require optimal function of the mitochondrial components, i.e., the solute carriers of various metabolites and associated enzymes, the electron transport chain machinery, the TOM/TIM mitochondrial protein import system, and proteins for iron and calcium homeostasis.

Using RNA-Seq analysis on isolated human islets, we have delineated the changes of such mitochondrial components following chronic exposure to different metabolic stresses hypothesized to participate to the etiology of type 2 diabetes, i.e., glucotoxicity, lipotoxicity, and glucolipotoxicity. This profiling, evaluated in light of previous studies, uncovered specific signatures of the different nutrients.

## 2. Materials and Methods

### 2.1. Reagents

Culture media, D-glucose, fatty acids, and other basic reagents were obtained from Sigma-Aldrich (St. Louis, MO, USA).

### 2.2. Human Islets and Treatments

Human islets were isolated from pancreases of deceased multiorgan donors (n = 8), who had provided written informed consent (ECIT consortium, http://ecit.dri-sanraffaele.org/, 2009). None of the donors were diagnosed with diabetes nor metabolic syndrome (clinical data are provided in Supplementary Table S1). Donors had an average BMI of 25.5 ± 2.2 kg/m^2^ and were aged 51.5 ± 7.6 years. Islets were maintained for a standard recovery period of time (between 1 to 4 days) in CMRL-1066 medium containing 5.5 mM glucose supplemented with 10% (vol./vol.) fetal calf serum and used for experiments straight away without shipping maneuver (isolation and experiments being performed in the same institution at the Hôpitaux Universitaires de Genève, Switzerland). Isolated islets were hand-picked, washed twice and further maintained for 3 days at physiological 5.5 mM glucose (G5.5 control) with either 0.4 mM oleate (unsaturated fatty acid C18:1, Olea) or 0.4 mM palmitate (saturated fatty acid C16:0, Palm). Islets were also exposed to high 25 mM glucose (G25) with 0.4 mM oleate (G25 + Olea), 0.4 mM palmitate (G25 + Palm), or the combination of both fatty acids at 0.4 mM (G25+Olea+Palm, 0.2 mM each); all conditions with 0.5% BSA in the culture medium [2]. Stock solutions of fatty acids were adjusted to 8 mM in 11% fatty acid-free BSA solution, without organic solvent, and stored at −20 °C as previously described [3]. This resulted in the addition of 0.55% BSA in the culture media supplemented with the tested fatty acids. The physiological 5.5 mM glucose in CMRL-1066 medium served as control. The different treatments were systematically performed in parallel cultures. Islet batches from different donors were used for transcript quantification using different techniques, those corresponding to donors #1–5 were used for RNA-Seq analyses, those of donors #1, 2, 4, 6–8 were used for quantitative RT-PCR, while those of donors #1, 2, and 6 were used for previously published NanoString^®^ (nCounter system, Seattle, WA, USA) technology analysis [4]; see Supplementary Table S1.

### 2.3. RNA-Sequencing

Total RNA was extracted from cultured isolated human islets with Trizol reagent (Invitrogen, Carlsbad, CA, USA) [4]. RNA sequencing was performed in Susanne Mandrup’s laboratory (University of Southern Denmark, Odense, Denmark) as detailed previously [2,5]. Accession numbers for each transcript in NCBI reference sequence format are provided in Supplementary Tables S2–S6.

### 2.4. Quantitative RT-PCR

An amount of 2 µg of isolated RNA was converted into cDNA as previously described [6]. Primers for SLC25A10, SLC25A11, SLC25A22, SLC8B1, TIMM13, and cyclophilin A (PPIA) were designed using the Primer Express Software (Applera Europe, Rotkreutz, Switzerland); see list of primers in Supplementary Table S7. Quantitative RT-PCR was performed for human islets from donors #1, #2, #4, #6, #7, #8 using a StepOnePlus^TM^ Real–Time PCR system (Thermo Fisher Scientific, Waltham, MA, USA). PCR products were quantified fluorometrically using the SYBR Green Master kit (Roche, Mannheim, Germany). Experiments were performed in duplicate for each transcript, and mean values were normalized to those of the reference mRNA cyclophilin A (PPIA).

### 2.5. Network Analysis

We selected specific human genes with a direct or indirect role in mitochondrial transport for network analysis (full transcriptome data not shown). Functional interaction networks were built using stringApp (v 1.4.2) in Cytoscape (v 3.6.1), which includes both physical interactions from experimental data and functional associations from curated pathways, automatic text mining, and prediction methods; with a confidence score cutoff of 0.4 [7]. Finally, transcriptomic data were assembled into a functional network using Omics Visualizer (v 1.3.0) in Cytoscape (3.8.1) [8]. To avoid inference of interdependence between gene expression levels and their functional links, the edges from the networks including transcriptomic data were removed.

### 2.6. Statistical Analysis

Significant changes were considered when two or more independent islet batches (donors) exhibited down or upregulation with a log_2_ fold change (log_2_ FC) threshold of 0.5 associated with at least one or more adjusted *p* < 0.05; highlighted in bold in the figures. For quantitative RT-PCR results, a mixed model approach for repeated measures was applied with a significance threshold of 0.05.

## 3. Results

**Figure 2:**
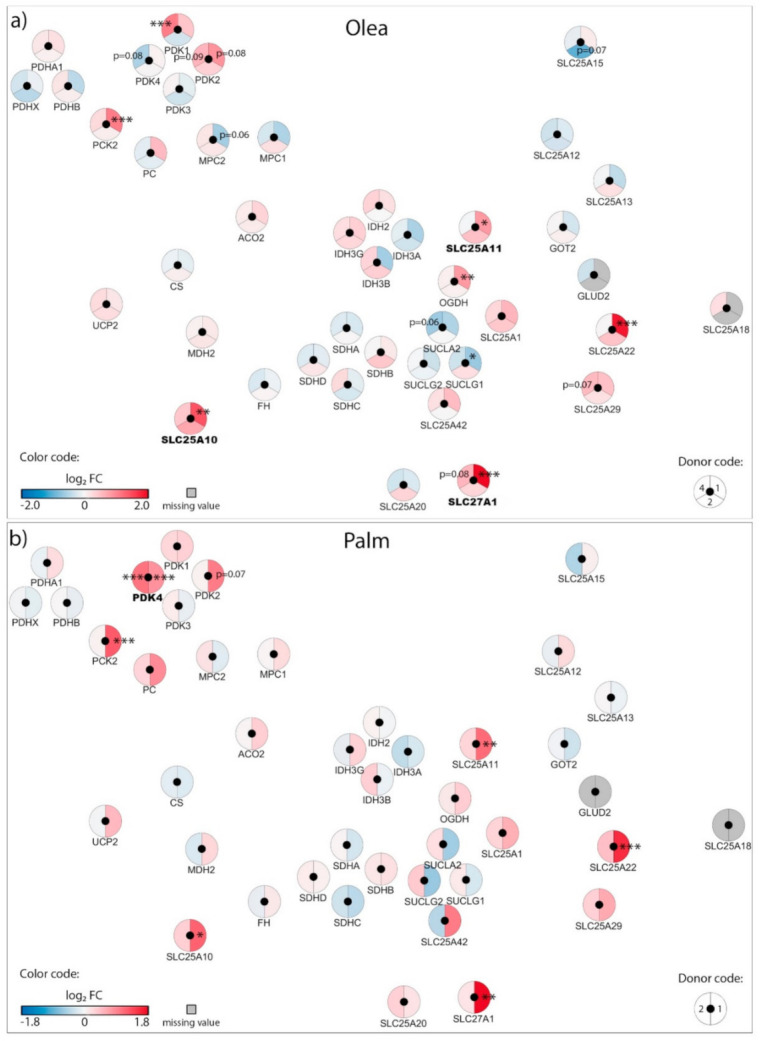
Effects of oleate (Olea) and palmitate (Palm) on the transcriptional regulation of mitochondrial solute carriers and associated genes: pyruvate metabolism, TCA cycle enzymes, amino acid metabolism and fatty acid transport. Human islets were exposed to 0.4 mM (**a**) Olea or (**b**) Palm at standard glucose concentration (G5.5) for 3 days before RNA-Seq analysis. Effects of lipotoxic culture conditions on transcript levels are compared to standard G5.5 medium and shown as upregulated (red), downregulated (blue), or unchanged (white). Missing values are represented in grey. Each disk is split into individual changes for the different islet donors. Color code reflects the transcriptional changes in log_2_ fold changes (log_2_ FC) for that particular gene in individual donors. Clinical data from individual donors are shown in Supplementary Table S1, while quantitative transcriptional data are shown in Supplementary Table S2. * adjusted *p* < 0.05, ** adjusted *p* < 0.01, *** adjusted *p* < 0.001 between control 5.5 mM glucose and the specific culture condition.

**Figure 3:**
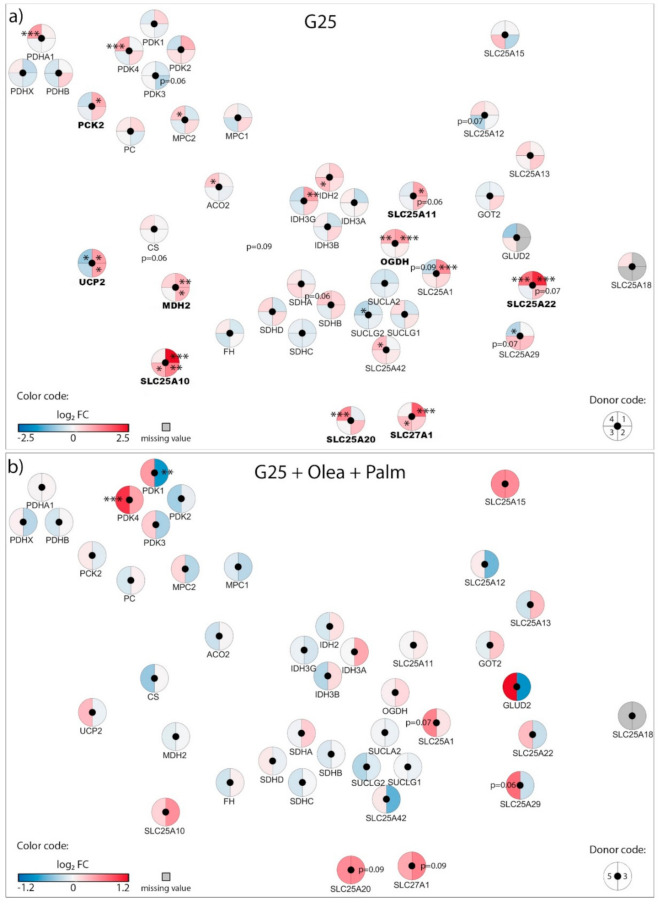
Effects of high 25 mM glucose (G25) and G25 plus 0.4 mM oleate and palmitate (G25 + Olea + Palm) on the transcriptional regulation of mitochondrial solute carriers and associated genes. Human islets were exposed to (**a**) G25 glucotoxic condition or (**b**) G25 + Olea + Palm glucolipotoxic condition for 3 days before RNA-Seq analysis. Effects of culture conditions on transcript levels are compared to standard G5.5 medium and shown as upregulated (red), downregulated (blue), or unchanged (white). Missing values are represented in grey. Each disk is split into individual changes for the different donors. Color code reflects the transcriptional changes in log_2_ fold changes (log_2_ FC) for that particular gene in individual donors. Clinical data from individual donors are shown in Supplementary Table S1, while quantitative transcriptional data are shown in Supplementary Table S2. * adjusted *p* < 0.05, ** adjusted *p* < 0.01, *** adjusted *p* < 0.001 between control 5.5 mM glucose and the specific culture condition.

**Figure 4:**
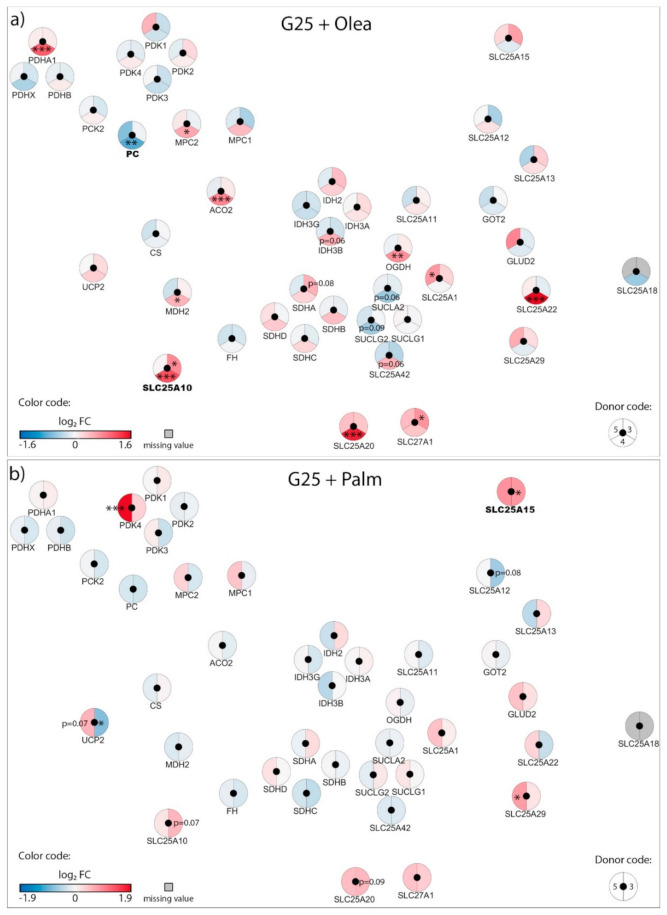
Effects of high 25 mM glucose (G25) plus 0.4 mM oleate (G25 + Olea) or palmitate (G25 + Palm) on the transcriptional regulation of mitochondrial solute carriers and associated genes. Human islets were exposed to (**a**) G25 + Olea or (**b**) G25 + Palm glucolipotoxic conditions for 3 days before RNA-Seq analysis. Effects of culture conditions on transcript levels are compared to standard G5.5 medium and shown as upregulated (red), downregulated (blue), or unchanged (white). Missing values are represented in grey. Each disk is split into individual changes for the different donors. Color code reflects the transcriptional changes in log_2_ fold changes (log_2_ FC) for that particular gene in individual donors. Clinical data from individual donors are shown in Supplementary Table S1, while quantitative transcriptional data are shown in Supplementary Table S2. * adjusted *p* < 0.05, ** adjusted *p* < 0.01, *** adjusted *p* < 0.001 between control 5.5 mM glucose and the specific culture condition.

**Figure 5:**
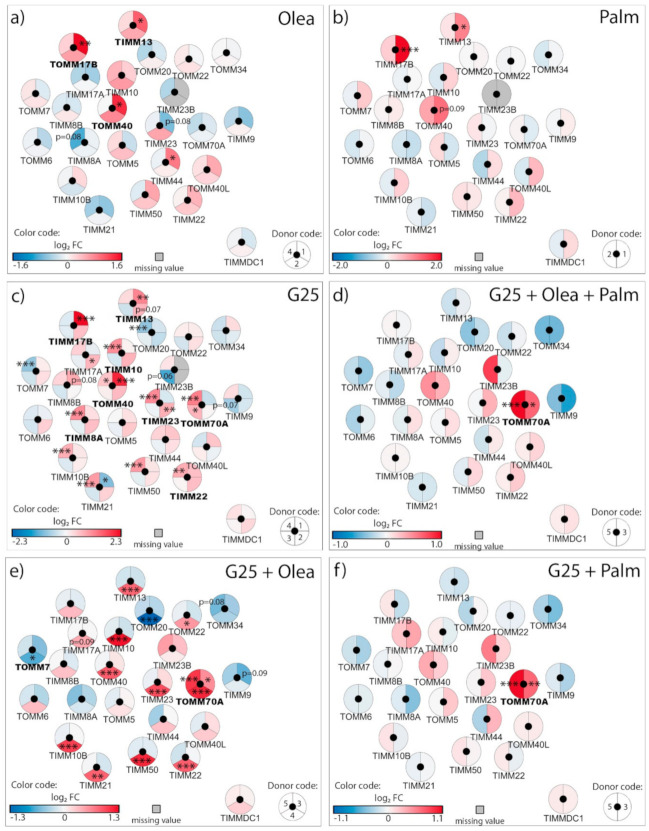
Effects of high 25 mM glucose (G25) and 0.4 mM oleate (Olea) or palmitate (Palm) on the transcriptional regulation of the outer and inner mitochondrial membrane translocases TOM/TIM. Human islets were exposed to (**a**) Olea at G5.5, (**b**) Palm at G5.5, (**c**) G25, (**d**) G25 + Olea + Palm, (**e**) G25 + Olea, and (**f**) G25 + Palm for 3 days before RNA-Seq analysis. Effects of culture conditions on transcript levels are compared to standard G5.5 medium and shown as upregulated (red), downregulated (blue), or unchanged (white). Missing values are represented in grey. Each disk is split into individual changes for the different donors. Color code reflects the transcriptional changes in log_2_ fold changes (log_2_ FC) for that particular gene in individual donors. * adjusted *p* < 0.05, ** adjusted *p* < 0.01, *** adjusted *p* < 0.001 between control 5.5 mM glucose and the specific culture condition.

Exposure of β-cells to chronic fuel surfeit triggers adaptive responses to cope with the increased insulin demand and also as a protective mechanism. When the adaptive mechanism fails, toxicity occurs with nutrient surplus, leading to β-cell dysfunction, dedifferentiation, and ultimately cell death. Although still debated, the terms lipotoxicity, glucotoxicity, and glucolipotoxicity are used to describe potentially nutrient-rich toxic conditions responsible for those pathogenic mechanisms in the context of type-2 diabetes. Of note, these terms may not only include potential toxic effects of nutrient excess, but also the beneficial adaptive mechanisms resulting from these conditions [9]. In previous reports, we characterized mitochondrion-associated genes in INS-1E β-cells using TaqMan Micro Fluidic Cards RT-PCR system (Thermo Fisher Scientific, Waltham, MA, USA) [10] and in human islets using NanoString^®^ targeted transcriptomic technology [4], revealing stress-specific signatures in response to chronic exposure to high glucose or fatty acids. Based on these studies and using an untargeted transcriptomic technology (RNA-Seq), we are now reporting the expression profile of mitochondrion transport-associated genes in response to specific metabolic stresses, i.e., to high glucose or fatty acids and also to their combination. This in vitro approach aims at mimicking, respectively, glucotoxic, lipotoxic, and glucolipotoxic conditions, potentially uncovering the specific contributions of the different stressors. We previously reported that such treatments induce marginal caspase-3 cleavage and essentially preserve β-cell differentiation, documented through the expression of the transcription factor IPF-1 [11]. Here, we provide a snapshot of the regulation of the mitochondrial carriers and associated genes from a whole-transcriptome data set (full data set not shown). We delineated a functional interaction network of selected genes using the STRING knowledgebase [7,12] (Figure 1b and Supplementary Figure S1). In particular, we selected mitochondrial metabolite carriers and associated genes involved in pyruvate metabolism, the tricarboxylic acid (TCA) cycle, amino acid metabolism and fatty acid transport; in the electron transport chain and related carriers; in the outer and inner mitochondrial membrane translocases TOM/TIM; in iron transport; and in calcium transport. Then, transcriptomic data were added into the functional gene network for visualization of each islet batch corresponding to the individual donors #1–5 (Figure 2, Figure 3, Figure 4 and Figure 5, Supplementary Figures S2 and S3, Supplementary Tables S2–S6). The main changes are summarized in Figure 7.

In order to compare alternative technologies commonly used for the assessment of mRNA levels, we measured some genes of interest with qRT-PCR (Supplementary Figure S4). This was performed on some of the islet batches used for the present RNA-Seq analysis (donors #1, #2, #4) and also on additional independent islet batches (donors #6–8). In addition to RNA-Seq and qRT-PCR, NanoString^®^ targeted transcriptomic technology was previously applied for delineation of mitochondrial transcriptome using material extracted from shared islet batches (donors #1, #2, #6); see Supplementary Table S1 and [4]. Collectively, this cross-comparison reveals important differences regarding relative mRNA levels, in particular for RNA-Seq versus qRT-PCR data. The latter requires amplification of a target sequence and use of an internal control, which confer limitations when measurements are performed on samples of various origins, such as islets isolated from different donors and not pooled for experimental in vitro treatments. This approach also shows that changes in gene expression in isolated human islets are highly batch specific.

### 3.1. Adaptation of Mitochondrial Solute Carriers Responsible for Metabolite Transport

Chronic effects of nutrient-rich metabolic stresses on mitochondrial carriers involved in the transport of pyruvate, TCA cycle intermediates, amino acids, fatty acids and associated genes were determined in isolated human islets at the end of a three-day exposure to the different diabetogenic milieus using RNA-Seq (Figure 2, Figure 3 and Figure 4, Supplementary Table S2).

In human islets cultured with physiological glucose G5.5, neither oleate nor palmitate modified expression of the pyruvate carrier (*MPC1* and *MPC2*) and the pyruvate dehydrogenase complex (*PDH*), see Figure 2a,b. However, treatment of islets with palmitate at G5.5 for 3 days increased the pyruvate dehydrogenase kinase 4 (*PDK4)* mRNA levels (Figure 2b), while a similar trend was observed for *PDK1* with oleate (Figure 2a). Oleate upregulated the dicarboxylate/malate carrier DIC (*SLC25A10*), the 2-oxoglutarate/malate carrier OGC (*SLC25A11*), and the fatty acid transport protein FATP1 (*SLC27A1*); while palmitate exhibited a similar pattern (Figure 2a,b), as observed previously using microarray analysis [13]. Quantitative RT-PCR analyses were performed on shared islet batches (donors #1, #2, and #4) as well as on additional batches (donors #6*–*8) for *SLC25A10*, *SLC25A11*, and *SLC25A22* (Supplementary Figure S4); confirming that variations in transcript levels are batch-specific. Overall, chronic exposure of islets to palmitate and oleate induced limited changes in the expression of genes of the mitochondrial solute carrier family.

High glucose (G25) increased expression of pyruvate dehydrogenase E1 subunit alpha 1 (*PDHA1*), pyruvate dehydrogenase kinase 4 (*PDK4*), and the pyruvate carrier (*MPC2*) in only one donor out of four (Figure 3a), suggesting that mitochondrial pyruvate handling should be preserved in these conditions. However, high glucose consistently upregulated the expression of the malate carrier DIC (*SLC25A10*), the 2-oxoglutarate/malate carrier OGC (*SLC25A11*), and the glutamate carrier GC1 (*SLC25A22*), along with the associated enzymes malate dehydrogenase 2 (*MDH2*) and 2-oxoglutarate dehydrogenase (*OGDH*). Of note, the uncoupling protein 2 (*UCP2*) was diversely modified in the different donors (Figure 3a). G25 also increased expression of the fatty acid transport protein FATP1 (*SLC27A1*) and the carnitine/acylcarnitine carrier CAC (*SLC25A20*). Such a profile suggests that glucotoxic conditions induce important mitochondrial anaplerotic/cataplerotic and NADPH-generating shuttle activities, as well as increased fatty acid import into mitochondria.

Regarding the glucolipotoxic conditions, expression of *PDK4* was unchanged at G25 plus oleate (Figure 4a), although significantly upregulated in one donor at G25 plus palmitate (Figure 4b) as well as G25 plus both saturated and unsaturated fatty acids (Figure 3b). This points to palmitate having a specific effect on *PDK4* expression, consistent with the increase in PDK4 we observed with palmitate alone at standard G5.5 (Figure 2a). Similar to G25 alone, G25 plus oleate upregulated the malate carrier DIC (*SLC25A10*) (Figure 4a). However, palmitate at G25, even combined with oleate, restored expression of DIC (*SLC25A10)* to nearly control levels (Figure 3b and Figure 4b, respectively). The expression of enzymes participating in the TCA cycle was marginally affected by glucolipotoxic conditions (Figure 3b and Figure 4a,b). Overall, chronic exposure of islets to high glucose plus palmitate and oleate induced moderate changes in the expression of genes of the mitochondrial solute carrier family, although exhibiting specific fatty acid signatures.

### 3.2. Adaptation of the Electron Transport Chain Machinery

Chronic effects of metabolic stress on the electron transport chain (ETC) machinery were then investigated in human islets at the end of the three-day exposure to the different diabetogenic milieus using RNA-Seq (Supplementary Figure S2, Supplementary Table S3). Treatment of islets with oleate at G5.5 for 3 days increased expression of the nuclear-encoded respiratory chain subunits of complex I (*NDUFS7*, *NDUFB7, NDUFS8*) and complex IV (*COX8A, COX5B*). Palmitate treatment, while inducing a similar trend, significantly upregulated complex V (*ATP5D*, *ATP5G1*). Expression of the phosphate carrier PiC (*SLC25A3*), the ADP/ATP translocases ANT1 and ANT2 (*SLC25A4* and *SLC25A5*) and the uncoupling proteins (*UCP2, SLC25A27* and *SLC25A14*) was preserved (Supplementary Figure S2a,b).

With respect to the other stressors, high glucose by itself caused the strongest expression changes in mitochondrial ETC subunits, inducing upregulation of the nuclear-encoded subunits of complex I (*NDUFS7, NDUFS8, NDUFB2, NDUFB9, NDUFB10*), complex III (*UQCRQ, UQCRC1, UQCRFS1, UQCR10*), complex IV (*COX5B*), complex V (*ATPIF1, ATP5J2*, *ATP5D, ATP5G1*), and downregulation of complex IV subunit (*COX7B*, *COX6C*), see Supplementary Figure S2c. High glucose also altered the expression of uncoupling proteins (*UCP2, SLC25A14*).

Addition of palmitate (alone or combined with oleate) at G25 elicited a general shift towards decreased expression of ETC subunits (Supplementary Figure S2d,f). This reduction in the overall G25 effect was less pronounced in the presence of oleate (Supplementary Figure S2e). In particular, islets from donor #4 exhibited strong changes in the expression of the different subunits of complexes I, III, IV and V, along with cytochrome C oxidase assembly components (Supplementary Figure S2e).

These results point to glucotoxic condition as the main effector for changes in the ETC machinery, while palmitate prevented most of the upregulations associated with high glucose.

### 3.3. Adaptation of the Translocases of the Outer and Inner Mitochondrial Membrane TOM/TIM

Next, we investigated mitochondrial membrane translocases responsible for preprotein import. Exposure of human islets to oleate, and to a lesser extent palmitate, at standard G5.5 for 3 days increased expression of the transmembrane channel of the outer membrane TOM complex *TOMM40*, the component of the intermembrane space TIM8–TIM13 complex *TIMM13*, and the component of the inner membrane TIM23 complex *TIMM17B* (Figure 5a,b; Supplementary Table S4).

Consistent with the changes observed in the ETC, high glucose caused the strongest transcriptional modifications in the translocases of the outer and inner mitochondrial membranes with respect to the other metabolic stresses. Similar to fatty acids at G5.5, G25 also per se upregulated *TOMM40, TIMM13*, and *TIMM17B,* while compared to fatty acids at G5.5 high glucose specifically upregulated the TOM70 receptor (*TOMM70A*) of the TOM complex, among other components of the inner membrane (*TIMM8A*, *TIMM10*, *TIMM22*, *TIMM23*), see Figure 5c. Some of the G25 effects were reinforced by the presence of fatty acids, in particular regarding the upregulation of the TOM70 receptor (Figure 5d–f). Complementary qRT-PCR analyses indicated that the upregulation of *TIMM13* by G25 is highly donor-dependent (Supplementary Figure S4). To our knowledge, this is the first description of transcriptional changes in the mitochondrial protein import machinery upon glucotoxic and lipotoxic conditions in pancreatic islets. Essentially, lipotoxicity and glucotoxicity increased expression of components of the outer membrane TOM complex, the intermembrane space TIM8–TIM13 complex and of the inner membrane TIM23 complex, while glucolipotoxicity strongly upregulated the *TOMM70A* receptor of the TOM complex.

### 3.4. Adaptation of the Mitochondrial Iron Transport

Iron is essential for mitochondrial redox activity through the heme synthesis pathway and, as a consequence, for the function of the ETC. Culture with oleate at G5.5 increased expression of the translocator *TSPO* and the ATP-binding cassette MITOSUR (*ABCB8*), while palmitate exhibited a similar pattern but additionally repressed expression of frataxin (*FXN*), see Supplementary Figure S3a,b and Supplementary Table S5. Interestingly, disruption of the frataxin gene in mice causes the loss of β-cell mass and then diabetes [14]. Similar to fatty acids, high glucose increased expression of *TSPO* and *ABCB8*, as well as the carrier CGI-69 (*SLC25A39*), which was marginally upregulated by oleate and palmitate. These results suggest shared responses between lipotoxic and glucotoxic conditions (Supplementary Figure S3c). Combination of G25 with oleate showed upregulation of mitoferrin-2 (*SLC25A28*), see Supplementary Figure S3E. Conversely, apoptosin (*SLC25A38*) was downregulated by the presence of fatty acids at G25 (Supplementary Figure S3e,f). Of note, mitochondrial ferritin (*FTMT*) was not detected in any of the donors upon any conditions. Overall, mitochondrial iron transport components exhibited moderate responses with high inter-individual variability upon diabetogenic conditions.

### 3.5. Adaptation of the Mitochondrial Calcium Transport

**Figure 6:**
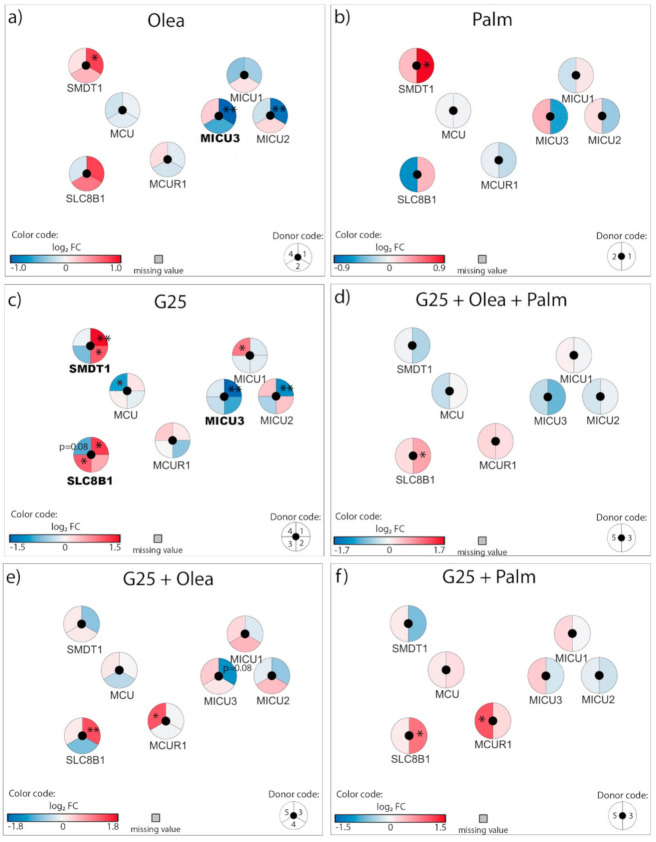
Effects of high 25 mM glucose (G25) and 0.4 mM oleate (Olea) or palmitate (Palm) on the transcriptional regulation of mitochondrial calcium transport genes. Human islets were exposed to (**a**) Olea at G5.5, (**b**) Palm at G5.5, (**c**) G25, (**d**) G25 + Olea + Palm, (**e**) G25 + Olea, and (**f**) G25 + Palm for 3 days before RNA-Seq analysis. Effects of culture conditions on transcript levels are compared to standard G5.5 medium and shown as upregulated (red), downregulated (blue), or unchanged (white). Missing values are represented in grey. Each disk is split into individual changes for the different donors. Color code reflects the transcriptional changes in log_2_ fold changes (log_2_ FC) for that particular gene in individual donors. * adjusted *p* < 0.05, ** adjusted *p* < 0.01 between control 5.5 mM glucose and the specific culture condition.

The next group of mitochondrial components we analyzed mediates calcium transport across the mitochondrial membrane. At G5.5, neither oleate nor palmitate modified expression of the Ca^2+^ uniporter *MCU*, while both fatty acids exhibited a trend for increased expression of the essential MCU regulator *SMDT1* (Figure 6a,b), an effect significantly induced by G25 alone (Figure 6c, Supplementary Table S6). Interestingly, the combination of high glucose with fatty acids counteracted the upregulation of *SMDT1* observed with individual stressors (Figure 6d–f). Oleate and G25 by themselves decreased expression of the MCU regulatory partners *MICU3*, while the combination of both nutrient-rich conditions blunted such effects (Figure 6a,c–e). High glucose also upregulated the Na^+^/Ca^2+^ exchanger NCLX (*SLC8B1*) (Figure 6c), an effect not revealed by qRT-PCR (Supplementary Figure S4).

Overall, some changes induced by either lipotoxic or glucotoxic conditions disappeared when glucolipotoxicity was applied, in particular the upregulation of *SMDT1* and the downregulation of MCU regulatory partners.

## 4. Discussion

### 4.1. Adaptation of Mitochondrial Solute Carriers Responsible for Metabolite Transport

We first analyzed the carriers and associated genes involved in mitochondrial metabolic fluxes. Of primary importance, pyruvate is the link between glycolysis and mitochondrial activation, allowing TCA cycle fueling. Upon diabetogenic conditions, the machinery for pyruvate entry and oxidation in mitochondria was essentially preserved, although palmitate increased the expression of pyruvate dehydrogenase kinase *PDK4*. In islets of non-obese βV59M diabetic mice, *PDK1* is strongly upregulated, at both RNA and protein levels, while TCA cycle enzymes are essentially downregulated [15]. Increased *PDK* expression may account for reduced PDH activity reported in ß-cells and pancreatic islets exposed to elevated fatty acids [16,17]. However, knockdown of both isoforms one and three of *PDK* in INS-1E β-cells does not affect metabolism–secretion coupling [18]. Interestingly, *PDK4* expression was specifically upregulated by the saturated fatty acid palmitate. Increased PDK4 activity inhibits PDH and rapidly suppresses mitochondrial pyruvate utilization. In mice fed a high-fat diet, upregulation of *PDK4* precedes diet-induced alteration of glucose oxidation in the heart [19], although we lack similar data on insulin-producing cells. These results suggest a specific fatty acid signature, resulting in lower pyruvate oxidation in the presence of saturated fat.

Downstream of pyruvate metabolism, intermediates of the TCA cycle are recruited to serve as substrates leading to the formation of coupling factors for metabolism–secretion coupling in β-cells [20]. Some of the diabetogenic conditions upregulated several mitochondrial carriers in human islets, i.e., the malate carrier DIC (*SLC25A10*), the α-ketoglutarate-malate exchanger OGC (*SLC25A11)*, and the glutamate carrier GC1 (*SLC25A22*). Despite some discrepancies between studies as well as mRNA versus protein levels [21], this observation suggests important mitochondrial anaplerotic/cataplerotic pathways and NAD(P)H-generating shuttle activities, thereby generating factors supporting glucose-stimulated insulin secretion [1,22,23,24,25]. Interestingly, the fatty acid transporter FATP1 was also upregulated by metabolic stresses, potentially promoting β-oxidation. In several models of obese type-2 diabetes, oxidation of fatty acids in β-cells is enhanced along with reduced glucose-stimulated insulin secretion [26,27,28], favoring detoxification of intracellular lipids. Consequently, channeling fatty acids towards β-oxidation in mitochondria would divert cytosolic fatty acids from the generation of lipid-derived amplifying signals for insulin exocytosis [22,29,30], possibly compensated by the glycerolipid/free fatty acid cycle [26].

Overall, nutrient-rich metabolic stresses induced (i) anaplerotic/cataplerotic machinery that preserves glucose oxidation and metabolism–secretion coupling, pointing to a mitohormetic response [31,32] and (ii) fatty acid import into mitochondria, diverting cytosolic fatty acids from the generation of amplification signals.

### 4.2. Adaptation of the Electron Transport Chain Machinery

The TCA cycle generates reducing equivalents transferred to the ETC by NADH and FADH_2_, promoting hyperpolarization of the mitochondrial membrane (∆Ψm) and ATP production, which is necessary for distal events on the plasma membrane inducing insulin exocytosis (Figure 1a).

Our results uncover increased expression of components of the ETC upon glucotoxic conditions, to a lesser extent upon lipotoxic conditions, and towards normalization with combined glucolipotoxicity. Regarding genes encoded by the mitochondrial genome, i.e., 13 ETC subunits, we previously reported that their expression is reduced in human islets upon high glucose with moderate effects upon oleate exposure [4,10]. Experiments with β-cell lines exposed to chronic high glucose or palmitate showed impaired mitochondrial hyperpolarization in response to glucose notwithstanding preserved mitochondrial respiration [11,33], pointing to changes in the mitochondrial inner membrane current–voltage relationship [33,34]. Conversely, and consistent with the present data, exposure of a clonal β-cell line to chronic high glucose results in elevated mitochondrial respiration, although glucose-stimulated insulin secretion is impaired [4,33]. These discrepancies between studies related to glucotoxicity could be explained by differences in the experimental conditions, such as the duration of the treatments (24/48/72 h). For the β-cell, glucose holds this apparent paradox of being its primary stimulus for metabolism–secretion coupling and becoming toxic when chronically reaching stimulatory concentrations. Palmitate combined with elevated glucose consistently reduces mitochondrial respiration and metabolism–secretion coupling [35]. This is in agreement with the notion that accumulation of glucose-derived malonyl-CoA accounts for adaptation to glucolipotoxicity [30], and is consistent with the blunted upregulation of ETC components we observed upon glucolipotoxic conditions.

When exploring the ETC machinery of pancreatic islets from type-2 diabetic donors, the observations are also variable. Anello et al. observed increased expression of complex I and complex V of the ETC with impaired hyperpolarization of ΔΨ_m_ and lower ATP production upon glucose stimulation [36]. Conversely, other studies have reported decreased expression of genes involved in the ETC [37,38], including isolated human islets exposed for 2 days to palmitate [39]. Rodent and cell culture studies support the finding that reduced expression of ETC components lowers oxidative phosphorylation (OXPHOS) enzyme activity and hence impairs glucose-stimulated ATP production and insulin exocytosis [40]. However, OXPHOS is the main upregulated set of genes in islets of a prediabetic mouse model in response to diabetogenic high-fat diet [41]. This observation could explain the high variability observed between the different studies [13,39] including ours, in the prediabetic state, enhanced OXPHOS by upregulation of the ETC could be a compensatory mechanism to increase ATP production and insulin exocytosis, while this mechanism is lost as diabetes progresses. Hence, different donors at different stages of diabetes with distinct β-cell functionality could show distinct responses.

### 4.3. Adaptation of the Translocases of the Outer and Inner Mitochondrial Membrane TOM/TIM

The concerted actions of TOM and TIM are responsible for the post-translational import of nuclear-encoded mitochondrial proteins [42]. Our unprecedented analysis of the TOM/TIM system by diabetogenic conditions in insulin-producing cells reveals upregulation of the TOM/TIM import machinery upon glucotoxic and lipotoxic conditions, potentially accounting for increased mitochondrial activity. Of note, glucolipotoxicity robustly and consistently upregulated *TOMM70A*. This receptor (TOM70) plays a key role in the import of mitochondrial carrier precursors [43]. Independently of its protein import function, TOM70 also sustains cell bioenergetics by mediating Ca^2+^ transfer from the endoplasmic reticulum (ER) to the mitochondria [44]. Elevation of mitochondrial Ca^2+^ concentrations favors the activity of Ca^2+^-sensitive mitochondrial dehydrogenases [45] and ATP synthase-dependent respiration in β-cells [46]. This observation is consistent with the increased ER–mitochondria contact sites in human islets and INS-1E cells exposed to glucotoxic conditions [47]. Despite the numerous ER–mitochondria contact sites, Ca^2+^ transfer into the mitochondria is impaired in these conditions [47], suggesting a possible compensatory mechanism by TOM70. Upregulated by glucotoxic conditions, *TIMM13* and *TIMM8A* form the Tom8–Tom13 complex that is involved in the import of the mitochondrial aspartate/glutamate carriers AGC1 (or aralar1) and AGC2 (or citrin) [48]. AGC1 is an important component of NADH shuttle activity in insulin-secreting cells, particularly solicited upon robust glycolytic flux [24,49]. In summary, chronic nutrient-rich metabolic stress increased transcription of key components of the TOM/TIM import machinery, potentially enhancing the mitochondrial oxidative phosphorylation activity.

### 4.4. Adaptation of the Mitochondrial Iron Transport

An increasing body of evidence suggests that iron accumulation is associated with elevated risk of type-2 diabetes and might be directly implicated in its pathophysiology [50,51,52,53,54,55]. In vitro, cellular iron import through the divalent metal transporter DMT1 is increased by diabetogenic conditions, such as exposure to cytokines [56] and glucolipotoxicity [57]. As a consequence, the cytosolic labile iron pool (LIP) is enlarged, favoring the production of reactive oxygen species (ROS) and impairing mitochondrial function [57]. Dietary iron restriction, or iron chelation, protects obese mice from alteration of β-cell function and diabetes [58]. Apart from its role in cytoplasmatic and nuclear functions, iron is necessary in mitochondria for the synthesis of heme and the associated iron sulfur cluster (ISC)-containing proteins of the ETC [59]. Iron is imported into mitochondria through mitoferrin 1 or 2 (*SLC25A37* or *SLC25A28*), stored by mitochondrial-ferritin (*FTMT*), and assembled into ISC with the help of frataxin (*FXN*) [60]. In contrast with one report documenting expression of *FTMT* in rodent islets [61], our RNA-Seq analysis did not detect mitochondrial ferritin in human islets. One can speculate that mitochondrial iron homeostasis in human islets is highly dynamic, with active frataxin-mediated ISC assembly and transfer to the ETC limiting its mitochondrial matrix storage [62,63]. We observed that palmitate repressed frataxin, favoring uncontrolled accumulation of mitochondrial iron. The role of *FXN* in the β-cell is illustrated by patients with Friedreich’s ataxia. These patients have reduced expression of *FXN* associated with β-cell demise secondary to oxidative stress-induced apoptosis [64] and with defects in insulin secretion and action [39]. Loss of frataxin impacts mitochondrial ATP production in vivo [65], consistent with its role in the building of the ETC. Of note, islets from type-2 diabetic donors have lower *FXN* expression levels [66].

Lipotoxicity and glucotoxicity increased expression of the ATP-binding cassette subfamily B member 8 (*ABCB8*) located in the inner mitochondrial membrane. There are no reports on the regulation of *ABCB8* upon such conditions, although its role in the export of mitochondrial iron and in the defense against oxidative stress has been documented [67,68]. ABCB8 forms a complex with other mitochondrial proteins, including succinate dehydrogenase, inorganic phosphate carrier, adenine nucleotide translocator, and ATP synthase [69]. As the mitochondrial respiratory chain is a major source of ROS in β-cells and other cell types [70,71,72], upregulation of *ABCB8* could represent a defense mechanism in β-cells. Finally, *SLC25A39* was significantly upregulated by high glucose and marginally by fatty acids. The function of *SLC25A39* is not yet well defined, although it is probably not directly implicated in iron transport. Indeed, its silencing does affect mitochondrial iron levels, while it impairs iron incorporation into heme [73], demonstrating a role for this gene in mitochondrial iron homeostasis.

Overall, past and present data show that diabetogenic conditions alter the expression of genes involved in mitochondrial iron homeostasis, with possible implications in the pathophysiology of diabetes and β-cell function.

### 4.5. Adaptation of the Mitochondrial Calcium Transport

When β-cells are stimulated with elevated glucose, mitochondrial ATP generation promotes plasma membrane depolarization and a subsequent rise in cytosolic Ca^2+^ concentration, thereby triggering insulin secretion. Part of the Ca^2+^ peak is transferred into mitochondria [74], as well as in the ER [75]. Increased mitochondrial Ca^2+^ enhances the activity of some dehydrogenases [76,77,78], promoting the generation of additive coupling factors for insulin exocytosis [79,80,81].

The channel responsible for mitochondrial Ca^2+^ uptake is the uniporter MCU and its expression is required in ß-cells for proper in vitro glucose-stimulated insulin secretion [82]. Consistent with previous reports [47], none of the tested conditions significantly modified *MCU* expression, in agreement with its complex post-translational regulation [83]. However, we show here that glucotoxicity upregulated *SMDT1* (essential MCU regulator, EMRE), which is indispensable for *MCU* activity [84]. Silencing of *MCU* in β-cells impairs the rise in mitochondrial Ca^2+^ evoked by cell depolarization and reduces the plateau phase of ATP/ADP ratio upon glucose stimulation without affecting mitochondrial membrane potential [85]. Accordingly, knockdown of *MCU* in mouse ß-cells inhibits glucose-evoked insulin exocytosis [86]. At the in vivo level, mice lacking MCU in β-cells display normal glycemia despite impaired glucose-stimulated insulin secretion, which was tested on isolated islets [82]. Surprisingly, whole-body *MCU* knockout mice exhibit a mild phenotype without noticeable impact on oxidative phosphorylation [87]. It has been proposed that cytosolic Ca^2+^, more than mitochondrial matrix Ca^2+^, may adapt OXPHOS to workload by adjusting the rate of pyruvate supply from the cytosol to the mitochondria [88]. Regarding Ca^2+^ efflux out of mitochondria, this is mediated by the Na^+^/Ca^2+^ exchanger NCLX (*SLC8B1*) [89] and its inhibition increases both mitochondrial Ca^2+^ concentration and glucose-stimulated insulin secretion [90]. In insulinoma cells, it was reported that high glucose abolishes the allosteric inhibition of NCLX, favoring Ca^2+^ efflux [91]. Here, we show that the glucotoxic condition upregulated *SLC8B1* (NCLX) in human islets. Therefore, NCLX could serve as a glucose sensor linking mitochondrial metabolism and Ca^2+^ signaling.

Overall, in human islets, a high glucose condition alters transcription of MCU regulatory partners and NCLX-mediating mitochondrial Ca^2+^ efflux, with a potential impact on mitochondrial activity and the generation of coupling factors.

## 5. Conclusions

**Figure 7:**
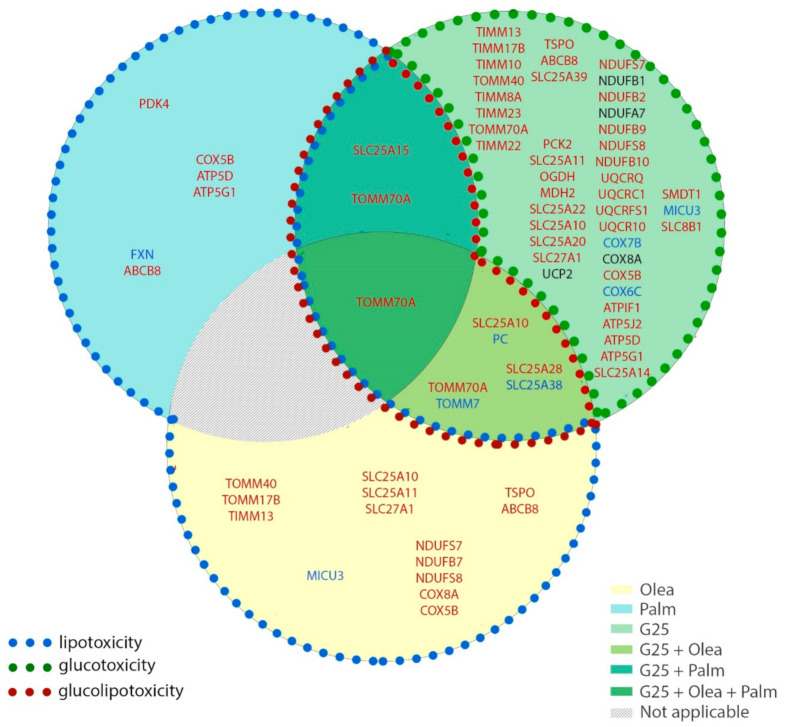
Stress-specific mitochondrial transcriptome profile of human islets upon lipotoxic, glucotoxic, and glucolipotoxic conditions. Isolated human islets were exposed for 3 days to different culture conditions: 25 mM glucose (G25) in the green circle, 0.4 mM oleate (Olea) in the yellow circle, and 0.4 mM palmitate (Palm) in the blue circle. The superimposition of the circles represents the mix of the corresponding conditions: G25 + Olea in the superimposition of the green and yellow circles, G25 + Palm in the superimposition of the green and blue circles, and G25 + Palm + Olea in the superimposition of the three conditions. The condition corresponding to Palm + Olea at G5.5 was not tested (not applicable). The circle sections contain the genes whose expression is modified upon that specific stress condition. Colors reflect positive (red), negative (blue) or variable (black) changes in mRNA. Significant changes were considered when two or more independent islet batches (donors) exhibited down or upregulation with a log_2_ FC threshold of 0.5 associated with one or more adjusted *p* < 0.05.

Lipotoxic, glucotoxic, and glucolipotoxic conditions applied to human islets induced specific expression changes in solute mitochondrial carriers and associated genes, TOM/TIM protein import machinery, ETC components, and iron and calcium transport. Glucotoxicity was the condition that altered the expression of the largest number of genes. Interestingly, the addition of fatty acids to the high glucose culture counteracted several of the changes, in particular regarding the ETC components and the TOM/TIM machinery (Figure 7). The specificities of changes could also discriminate between saturated and unsaturated fatty acids. Delineation of nutrient-specific signatures in human islets may provide novel insights for the management of type-2 diabetes.

## Supplementary Materials

The following are available online at https://www.mdpi.com/2218-273X/10/11/1543/s1; Supplementary Figure S1. Functional interaction network; Supplementary Figure S2. Transcriptional regulation of the electron transport chain machinery and related mitochondrial carriers in human islets; Supplementary Figure S3. Transcriptional regulation of mitochondrial iron transport genes in human islets; Supplementary Figure S4: Effects of high 25 mM glucose (G25) and 0.4 mM palmitate (Palm) or oleate (Olea) on mRNA levels of selected genes in human islets measured by quantitative RT-PCR, normalized to cyclophilin A (PPIA). See Supplementary Table S1 for details on the donors. Supplementary Table S1: Clinical data of the human donors of pancreatic islets; Supplementary Table S2. Quantitative data related to the transcriptomic profiles of mitochondrial solute carriers and associated genes in human islets upon metabolic stress; Supplementary Table S3. Quantitative data related to the transcriptomic profiles of the electron transport chain machinery and related mitochondrial carriers in human islets upon metabolic stress; Supplementary Table S4. Quantitative data related to the transcriptomic profiles of the outer and inner mitochondrial membrane translocases TOM/TIM machinery in human islets upon metabolic stress; Supplementary Table S5. Quantitative data related to the transcriptomic profiles of mitochondrial iron transport genes in human islets under metabolic stress; Supplementary Table S6. Quantitative data related to the transcriptomic profiles of mitochondrial calcium transport genes in human islets upon metabolic stress. Supplementary Table S7: Primers used for quantitative RT-PCR analysis.
