# Supplementary material for: Mitochondrial Carriers Regulating Insulin Secretion Profiled in Human Islets upon Metabolic Stress
Source: Biomolecules. 2020 Nov 12;10(11):1543. doi: 10.3390/biom10111543 (PMC7697104; doi:10.3390/biom10111543)
Supplement: Supplementary file 1 [file biomolecules-10-01543-s001.pdf]

Jiménez-Sánchez *et al*. **Supplementary files**

Mitochondrial carriers regulating insulin secretion profiled in human islets upon metabolic stress

Supplementary Table S1: Clinical Data of the human donors of pancreatic islets, type of analyses performed and tested conditions.

Supplementary Table S2: Quantitative data related to the transcriptomic profiles of mitochondrial solute carriers and associated genes in human islets upon metabolic stress. NA: Not applicable, ND: not detected.

Supplementary Table S3: Quantitative data related to the transcriptomic profiles of the electron transport chain machinery and related mitochondrial carriers in human islets upon metabolic stress.NA: Not applicable, ND: not detected.

Supplementary Table S4: Quantitative data related to the transcriptomic profiles of the outer and inner mitochondrial membrane translocases TOM/TIM machinery in human islets upon metabolic stress.NA: Not applicable, ND: not detected.

Supplementary Table S5: Quantitative data related to the transcriptomic profiles of mitochondrial iron transport genes in human islets under metabolic stress. NA: Not applicable, ND: not detected.

Supplementary Table S6: Quantitative data related to the transcriptomic profiles of mitochondrial calcium transport genes in human islets upon metabolic stress. NA: Not applicable, ND: not detected.

Supplementary Table S7: Primers used for quantitative RT-PCR analysis

Supplementary Figure S1: Functional interaction network of human (**a**) mitochondrial calcium transport genes; (**b**) outer and inner mitochondrial membrane translocases TOM/TIM machinery; (**c**) electron transport chain machinery and related carriers; (**d**) mitochondrial iron transport genes. Nodes were connected using the STRING interaction knowledgebase with a confidence score >0.4.

Supplementary Figure S2: Effects of high 25 mM glucose (G25) and 0.4 mM oleate (Olea) or palmitate (Palm) on the transcriptional regulation of the electron transport chain machinery. Human islets were exposed to (a) Olea at G5.5, (b) Palm at G5.5, (c) G25, (d) G25+Olea+Palm, (e) G25+Olea, and (d) G25+Palm for 3 days before RNA-Seq analysis. Effects of culture conditions on transcript levels are compared to standard G5.5 medium and shown as upregulated (red), downregulated (blue), or unchanged (white). Missing values are represented in grey. Each disk is split into individual changes for the

different donors. Color code reflects the transcriptional changes in log2 fold changes (log2 FC) for that particular gene in individual donors. *adjusted p < 0.05, **adjusted p < 0.01, ***adjusted p < 0.001 between control 5.5 mM glucose and the specific culture condition.

Supplementary Figure S3: Effects of high 25 mM glucose (G25) and 0.4 mM oleate (Olea) or palmitate (Palm) on the transcriptional regulation of mitochondrial iron transport genes. Human islets were exposed to (**a**) Olea at G5.5, (**b**) Palm at G5.5, (**c**) G25, (**d**) G25+Olea+Palm, (**e**) G25+Olea, and (f) G25+Palm for 3 days before RNA-Seq analysis. Effects of culture conditions on transcript levels are compared to standard G5.5 medium and shown as upregulated (red), downregulated (blue), or unchanged (white). Missing values are represented in grey. Each disk is split into individual changes for the different donors. Color code reflects the transcriptional changes in $\log_2$ fold changes ($\log_2$ FC) for that particular gene in individual donors. *adjusted p < 0.05, **adjusted p < 0.01, ***adjusted p < 0.001 between control 5.5 mM glucose and the specific culture condition.

Supplementary Figure S4: Effects of high 25 mM glucose (G25) and 0.4 mM oleate (Olea) or palmitate (Palm) on the mRNA levels of selected genes in human islets measured by quantitative RT–PCR, normalized to cyclophilin A (*PPIA*).

Supplementary Table S1: Clinical Data of the human donors of pancreatic islets, type of analyses performed and tested conditions (25 mM glucose, G25; 0.4 mM oleate, Olea; 0.4 mM palmitate, Palm; 0.2 mM oleate plus 0.2 mM palmitate, Olea+Palm). * DOI: 10.1093/hmg/ddv247

Donor	Gender	Age (years)	BMI (kg/m^2)	Cause of death	Culture (days)	Viability (%)	Purity (%)	Type of analysis performed	Tested conditions in RNA-Seq analyses
#1	M	56	28.6	Crania trauma	3	90	50	RNA–Seq, qRT–PCR, and previously published* NanoString®	Olea, Palm, G25
#2	M	59	27.2	Trauma	2	90	60	RNA–Seq, Q–RT–PCR, and previously published* NanoString®	Olea, Palm, G25
#3	F	41	22.4	Cerebral haemorrage	2	90	80	RNA–Seq	G25, G25+Olea+Palm, G25+Olea, G25+Palm
#4	M	46	27.2	Cerebral haemorrage	2	90	75	RNA–Seq, qRT–PCR	G25, G25+Olea
#5	F	59	23.7	Cerebral trauma	4	90	84	RNA–Seq	G25+Olea+Palm, G25+Olea, G25+Palm
#6	M	43	23.2	Crania trauma	1	95	87	qRT–PCR, and previously published* NanoString®	
#7	M	59	25.6	Cerebral haemorrage	3	90	75	qRT–PCR	
#8	M	49	26.2	Cerebral haemorrage	5	90	84	qRT–PCR	
M (6)	75%	52.0 ± 6.9	26.3 ± 1.8		2.7 ± 1.3	90.8 ± 2.0	71.8 ±14.2		

F (2)	25%	50.0 ± 12.7	23.1 ± 0.9	3.0 ± 1.4	90 ± 0	82.0 ±2.8
All (8)	100%	51.5 ± 7.6	25.5 ± 2.2	2.8 ± 1.3	90.6 ± 1.80	74.3 ±13.0

Supplementary Table S2. Quantitative data related to the transcriptomic profiles of mitochondrial solute carriers and associated genes in human islets upon metabolic stress. NA: Not applicable (condition not tested); ND: not detected.

			Donor #1		Donor #2		Donor #3		Donor #4		Donor #5	
	Accession (NCBI Ref. Sequence)	Gene symbol	**Log_2 FC**	**Adjusted *p***	**Log_2 FC**	**Adjusted *p***	**Log_2 FC**	**Adjusted *p***	**Log_2 FC**	**Adjusted *p***	**Log_2 FC**	**Adjusted *p***
Olea	NM_001098	ACO2	0.341	0.425	0.123	0.858	NA	NA	0.115	0.831	NA	NA
	NM_004077	CS	-0.134	0.760	0.097	0.882	NA	NA	-0.089	0.880	NA	NA
	NM_000143	FH	-0.081	0.890	0.047	0.965	NA	NA	-0.211	0.643	NA	NA
	NM_012084	GLUD2	ND	ND	ND	ND	NA	NA	-0.307	0.816	NA	NA
	NM_002080	GOT2	-0.259	0.493	0.079	0.913	NA	NA	0.031	0.975	NA	NA
	NM_002168	IDH2	0.219	0.642	-0.009	1.000	NA	NA	0.351	0.385	NA	NA
	NM_005530	IDH3A	-0.552	0.123	-0.285	0.557	NA	NA	-0.132	0.801	NA	NA
	NM_174855	IDH3B	-0.586	0.157	0.386	0.443	NA	NA	0.323	0.429	NA	NA
	NM_174869	IDH3G	0.371	0.502	0.290	0.590	NA	NA	0.241	0.584	NA	NA
	NM_001282404	MDH2	0.139	0.755	0.180	0.728	NA	NA	0.063	0.923	NA	NA
	NM_016098	MPC1	-0.525	0.214	0.221	0.733	NA	NA	-0.242	0.590	NA	NA
	NM_001143674	MPC2	-0.591	0.066	0.161	0.771	NA	NA	0.170	0.712	NA	NA
	NM_001165036	OGDH	0.864	0.006	0.069	0.930	NA	NA	0.081	0.891	NA	NA
	NM_000920	PC	0.559	0.410	-0.152	0.875	NA	NA	-0.157	0.772	NA	NA
	NM_004563	PCK2	1.107	0.001	0.093	0.895	NA	NA	0.270	0.508	NA	NA
	NM_001173456	PDHA1	0.218	0.636	0.071	0.935	NA	NA	0.165	0.733	NA	NA
	NM_001173468	PDHB	-0.428	0.248	0.093	0.899	NA	NA	0.178	0.714	NA	NA
	NM_003477	PDHX	-0.101	0.864	-0.443	0.349	NA	NA	-0.269	0.523	NA	NA
	NM_002610	PDK1	0.456	0.464	-0.269	0.704	NA	NA	1.106	0.000	NA	NA
	NM_001199898	PDK2	0.910	0.080	0.362	0.517	NA	NA	0.610	0.088	NA	NA
	NM_001142386	PDK3	-0.132	0.761	-0.299	0.501	NA	NA	0.066	0.915	NA	NA
	NM_002612	PDK4	0.051	0.934	0.038	0.972	NA	NA	-0.544	0.077	NA	NA
	NM_001294332	SDHA	-0.219	0.627	0.026	0.995	NA	NA	-0.189	0.679	NA	NA

	NM_003000	SDHB	0.140	0.801	0.444	0.308	NA	NA	0.011	1.000	NA	NA
	NM_003001	SDHC	-0.148	0.795	-0.231	0.704	NA	NA	0.267	0.538	NA	NA
	NM_001276503	SDHD	-0.169	0.728	0.165	0.793	NA	NA	-0.190	0.697	NA	NA
	NM_005984	SLC25A1	0.611	0.146	0.435	0.320	NA	NA	0.292	0.450	NA	NA
	NM_012140	SLC25A10	1.458	0.005	0.746	0.108	NA	NA	0.435	0.252	NA	NA
	NM_001165418	SLC25A11	0.857	0.042	0.509	0.267	NA	NA	-0.036	0.968	NA	NA
	NM_003705	SLC25A12	-0.185	0.737	-0.291	0.584	NA	NA	-0.301	0.480	NA	NA
	NM_001160210	SLC25A13	-0.396	0.334	0.216	0.730	NA	NA	-0.045	0.962	NA	NA
	NM_014252	SLC25A15	0.119	0.955	-1.011	0.066	NA	NA	-0.130	0.886	NA	NA
	NM_001303484	SLC25A18	ND	ND	ND	ND	NA	NA	0.226	0.817	NA	NA
	NM_000387	SLC25A20	-0.210	0.731	0.330	0.567	NA	NA	-0.276	0.535	NA	NA
	NM_001191061	SLC25A22	1.905	0.000	0.496	0.240	NA	NA	0.030	0.975	NA	NA
	NM_001039355	SLC25A29	0.508	0.298	0.216	0.731	NA	NA	0.565	0.075	NA	NA
	NM_178526	SLC25A42	0.543	0.462	0.015	1.000	NA	NA	0.224	0.637	NA	NA
	NM_198580	SLC27A1	2.010	0.000	0.310	0.621	NA	NA	0.608	0.078	NA	NA
	NM_003850	SUCLA2	-0.514	0.162	-0.009	1.000	NA	NA	-0.632	0.057	NA	NA
	NM_003849	SUCLG1	-0.662	0.042	0.229	0.655	NA	NA	-0.302	0.442	NA	NA
	NM_001177599	SUCLG2	-0.323	0.580	-0.076	0.948	NA	NA	-0.025	0.994	NA	NA
	NM_003355	UCP2	0.167	0.829	0.252	0.685	NA	NA	0.257	0.619	NA	NA
Palm	NM_001098	ACO2	0.334	0.455	0.037	1.000	NA	NA	NA	NA	NA	NA
	NM_004077	CS	-0.159	0.741	-0.139	1.000	NA	NA	NA	NA	NA	NA
	NM_000143	FH	0.130	0.818	-0.111	1.000	NA	NA	NA	NA	NA	NA
	NM_012084	GLUD2	ND	ND	ND	ND	NA	NA	NA	NA	NA	NA
	NM_002080	GOT2	-0.258	0.524	-0.062	1.000	NA	NA	NA	NA	NA	NA
	NM_002168	IDH2	-0.038	1.000	0.045	1.000	NA	NA	NA	NA	NA	NA
	NM_005530	IDH3A	-0.199	0.681	-0.347	1.000	NA	NA	NA	NA	NA	NA
	NM_174855	IDH3B	-0.076	0.939	0.334	1.000	NA	NA	NA	NA	NA	NA
	NM_174869	IDH3G	0.309	0.618	-0.122	1.000	NA	NA	NA	NA	NA	NA

	NM_001282404	MDH2	0.256	0.524	-0.182	1.000	NA	NA	NA	NA	NA	NA
	NM_016098	MPC1	0.229	0.669	0.041	1.000	NA	NA	NA	NA	NA	NA
	NM_001143674	MPC2	-0.151	0.758	0.181	1.000	NA	NA	NA	NA	NA	NA
	NM_001165036	OGDH	0.342	0.426	0.131	1.000	NA	NA	NA	NA	NA	NA
	NM_000920	PC	0.856	0.156	0.281	1.000	NA	NA	NA	NA	NA	NA
	NM_004563	PCK2	1.223	0.000	0.053	1.000	NA	NA	NA	NA	NA	NA
	NM_001173456	PDHA1	0.217	0.673	-0.080	1.000	NA	NA	NA	NA	NA	NA
	NM_001173468	PDHB	-0.113	0.851	0.014	1.000	NA	NA	NA	NA	NA	NA
	NM_003477	PDHX	-0.124	0.857	-0.057	1.000	NA	NA	NA	NA	NA	NA
	NM_002610	PDK1	0.320	0.681	0.254	1.000	NA	NA	NA	NA	NA	NA
	NM_001199898	PDK2	0.976	0.066	0.045	1.000	NA	NA	NA	NA	NA	NA
	NM_001142386	PDK3	-0.103	0.851	0.092	1.000	NA	NA	NA	NA	NA	NA
	NM_002612	PDK4	0.801	0.004	1.039	0.004	NA	NA	NA	NA	NA	NA
	NM_001294332	SDHA	-0.225	0.663	0.043	1.000	NA	NA	NA	NA	NA	NA
	NM_003000	SDHB	0.195	0.724	0.136	1.000	NA	NA	NA	NA	NA	NA
	NM_003001	SDHC	-0.371	0.466	-0.261	1.000	NA	NA	NA	NA	NA	NA
	NM_001276503	SDHD	0.060	0.959	0.088	1.000	NA	NA	NA	NA	NA	NA
	NM_005984	SLC25A1	0.591	0.184	0.295	1.000	NA	NA	NA	NA	NA	NA
	NM_012140	SLC25A10	1.161	0.045	0.329	1.000	NA	NA	NA	NA	NA	NA
	NM_001165418	SLC25A11	1.096	0.006	0.279	1.000	NA	NA	NA	NA	NA	NA
	NM_003705	SLC25A12	0.243	0.639	-0.102	1.000	NA	NA	NA	NA	NA	NA
	NM_001160210	SLC25A13	-0.082	0.929	0.009	1.000	NA	NA	NA	NA	NA	NA
	NM_014252	SLC25A15	0.071	0.970	-0.446	1.000	NA	NA	NA	NA	NA	NA
	NM_001303484	SLC25A18	ND	ND	ND	ND	NA	NA	NA	NA	NA	NA
	NM_000387	SLC25A20	0.164	0.819	0.319	1.000	NA	NA	NA	NA	NA	NA
	NM_001191061	SLC25A22	1.544	0.000	0.456	1.000	NA	NA	NA	NA	NA	NA
	NM_001039355	SLC25A29	0.628	0.195	0.322	1.000	NA	NA	NA	NA	NA	NA
	NM_178526	SLC25A42	0.985	0.114	-0.403	1.000	NA	NA	NA	NA	NA	NA

	NM_198580	SLC27A1	1.759	0.002	0.151	1.000	NA	NA	NA	NA	NA	NA
	NM_003850	SUCLA2	-0.534	0.152	0.199	1.000	NA	NA	NA	NA	NA	NA
	NM_003849	SUCLG1	-0.202	0.658	0.115	1.000	NA	NA	NA	NA	NA	NA
	NM_001177599	SUCLG2	-0.586	0.268	0.366	1.000	NA	NA	NA	NA	NA	NA
	NM_003355	UCP2	0.506	0.413	-0.048	1.000	NA	NA	NA	NA	NA	NA
G25	NM_001098	ACO2	0.080	0.918	-0.147	0.811	-0.069	0.938	0.557	0.036	NA	NA
	NM_004077	CS	0.040	0.954	-0.014	1.000	0.031	0.987	0.238	0.436	NA	NA
	NM_000143	FH	-0.270	0.483	0.019	1.000	-0.368	0.374	0.149	0.671	NA	NA
	NM_012084	GLUD2	0.492	0.184	0.283	0.541	0.654	0.022	0.173	0.630	NA	NA
	NM_002080	GOT2	-0.480	0.154	0.075	0.923	0.012	1.000	0.134	0.702	NA	NA
	NM_002168	IDH2	-0.374	0.362	0.392	0.395	-0.118	0.858	0.110	0.780	NA	NA
	NM_005530	IDH3A	1.019	0.006	0.435	0.316	-0.242	0.613	-0.436	0.149	NA	NA
	NM_174855	IDH3B	0.766	0.006	0.757	0.010	0.040	0.976	0.405	0.139	NA	NA
	NM_174869	IDH3G	1.025	0.001	0.140	0.782	0.032	0.986	0.812	0.001	NA	NA
	NM_001282404	MDH2	1.215	0.000	0.366	0.383	0.163	0.767	-0.469	0.093	NA	NA
	NM_016098	MPC1	2.496	0.000	1.388	0.000	0.796	0.011	0.120	0.772	NA	NA
	NM_001143674	MPC2	0.941	0.018	0.705	0.064	-0.090	0.906	-0.221	0.489	NA	NA
	NM_001165036	OGDH	-0.065	0.899	0.170	0.819	0.293	0.687	0.673	0.015	NA	NA
	NM_000920	PC	-0.343	0.338	-0.106	0.873	-0.191	0.708	-0.323	0.293	NA	NA
	NM_004563	PCK2	-0.306	0.370	0.222	0.629	-0.232	0.625	0.289	0.335	NA	NA
	NM_001173456	PDHA1	-0.273	0.649	-0.140	0.894	-0.224	0.677	-0.593	0.056	NA	NA
	NM_001173468	PDHB	0.104	0.869	-0.039	0.983	-0.628	0.073	0.324	0.297	NA	NA
	NM_003477	PDHX	0.062	0.932	0.522	0.218	0.079	0.932	0.389	0.198	NA	NA
	NM_002610	PDK1	ND	ND	ND	ND	ND	ND	0.242	0.661	NA	NA
	NM_001199898	PDK2	0.370	0.681	-0.265	0.749	-0.034	0.989	0.170	0.631	NA	NA
	NM_001142386	PDK3	0.859	0.013	0.525	0.136	-0.150	0.823	-0.429	0.136	NA	NA
	NM_002612	PDK4	0.102	0.852	-0.006	1.000	-0.072	0.931	1.058	0.000	NA	NA
	NM_001294332	SDHA	0.010	1.000	0.453	0.228	-0.310	0.441	-0.331	0.281	NA	NA

	NM_003000	SDHB	-0.518	0.174	-0.284	0.577	-0.174	0.761	0.148	0.670	NA	NA
	NM_003001	SDHC	0.411	0.494	-0.142	0.922	-0.011	1.000	-0.239	0.539	NA	NA
	NM_001276503	SDHD	0.818	0.124	0.231	0.710	0.265	0.589	-0.425	0.217	NA	NA
	NM_005984	SLC25A1	-0.264	0.423	-0.595	0.061	0.022	0.995	-0.033	0.942	NA	NA
	NM_012140	SLC25A10	-0.050	0.926	0.352	0.320	-0.194	0.689	0.893	0.000	NA	NA
	NM_001165418	SLC25A11	-0.143	0.809	0.361	0.495	-0.413	0.285	0.149	0.673	NA	NA
	NM_003705	SLC25A12	-0.283	0.406	0.328	0.384	-0.219	0.640	0.523	0.050	NA	NA
	NM_001160210	SLC25A13	ND	ND	ND	ND	0.210	0.902	-0.799	0.337	NA	NA
	NM_014252	SLC25A15	-0.094	0.827	0.392	0.279	-0.105	0.844	-0.170	0.603	NA	NA
	NM_001303484	SLC25A18	2.211	0.000	0.622	0.077	-0.121	0.849	1.512	0.000	NA	NA
	NM_000387	SLC25A20	0.014	1.000	-0.593	0.390	0.671	0.213	-0.050	0.948	NA	NA
	NM_001191061	SLC25A22	-0.006	1.000	0.654	0.100	0.631	0.076	-0.580	0.034	NA	NA
	NM_001039355	SLC25A29	1.728	0.002	0.479	0.358	0.704	0.044	0.070	0.881	NA	NA
	NM_178526	SLC25A42	-0.149	0.795	0.688	0.110	0.026	0.995	1.288	0.000	NA	NA
	NM_198580	SLC27A1	-0.128	0.780	0.195	0.702	0.350	0.357	0.196	0.543	NA	NA
	NM_003850	SUCLA2	0.405	0.259	0.365	0.371	-0.206	0.677	0.519	0.059	NA	NA
	NM_003849	SUCLG1	-0.243	0.615	-0.193	0.766	-0.212	0.675	-0.124	0.744	NA	NA
	NM_001177599	SUCLG2	-0.316	0.412	0.333	0.449	-0.125	0.843	0.369	0.221	NA	NA
	NM_003355	UCP2	1.016	0.025	0.950	0.012	-0.534	0.179	-0.823	0.017	NA	NA
G25+Olea+Palm	NM_001098	ACO2	NA	NA	NA	NA	0.019	0.995	NA	NA	-0.187	0.895
	NM_004077	CS	NA	NA	NA	NA	0.011	1.000	NA	NA	-0.389	0.465
	NM_000143	FH	NA	NA	NA	NA	0.048	0.965	NA	NA	-0.168	0.921
	NM_012084	GLUD2	NA	NA	NA	NA	-0.821	0.553	NA	NA	1.161	0.292
	NM_002080	GOT2	NA	NA	NA	NA	0.267	0.545	NA	NA	-0.086	0.997
	NM_002168	IDH2	NA	NA	NA	NA	0.126	0.831	NA	NA	-0.133	0.963
	NM_005530	IDH3A	NA	NA	NA	NA	0.426	0.227	NA	NA	0.004	1.000
	NM_174855	IDH3B	NA	NA	NA	NA	0.154	0.800	NA	NA	-0.279	0.730
	NM_174869	IDH3G	NA	NA	NA	NA	-0.154	0.800	NA	NA	-0.055	1.000

NM_001282404	MDH2	NA	NA	NA	NA	-0.017	0.996	NA	NA	-0.084	0.997
NM_016098	MPC1	NA	NA	NA	NA	-0.265	0.577	NA	NA	-0.120	0.977
NM_001143674	MPC2	NA	NA	NA	NA	-0.278	0.517	NA	NA	0.172	0.899
NM_001165036	OGDH	NA	NA	NA	NA	0.166	0.770	NA	NA	0.052	1.000
NM_000920	PC	NA	NA	NA	NA	0.045	0.974	NA	NA	-0.132	0.984
NM_004563	PCK2	NA	NA	NA	NA	-0.101	0.903	NA	NA	0.078	1.000
NM_001173456	PDHA1	NA	NA	NA	NA	0.032	0.980	NA	NA	0.021	1.000
NM_001173468	PDHB	NA	NA	NA	NA	0.014	1.000	NA	NA	-0.148	0.931
NM_003477	PDHX	NA	NA	NA	NA	-0.262	0.581	NA	NA	0.044	1.000
NM_002610	PDK1	NA	NA	NA	NA	-0.779	0.006	NA	NA	0.499	0.240
NM_001199898	PDK2	NA	NA	NA	NA	-0.076	0.935	NA	NA	-0.338	0.649
NM_001142386	PDK3	NA	NA	NA	NA	-0.326	0.411	NA	NA	0.234	0.784
NM_002612	PDK4	NA	NA	NA	NA	0.453	0.166	NA	NA	0.992	0.000
NM_001294332	SDHA	NA	NA	NA	NA	0.226	0.638	NA	NA	-0.043	1.000
NM_003000	SDHB	NA	NA	NA	NA	-0.011	1.000	NA	NA	-0.131	0.963
NM_003001	SDHC	NA	NA	NA	NA	-0.013	1.000	NA	NA	-0.153	0.932
NM_001276503	SDHD	NA	NA	NA	NA	-0.060	0.946	NA	NA	0.082	1.000
NM_005984	SLC25A1	NA	NA	NA	NA	0.113	0.860	NA	NA	0.616	0.071
NM_012140	SLC25A10	NA	NA	NA	NA	0.554	0.136	NA	NA	0.203	0.889
NM_001165418	SLC25A11	NA	NA	NA	NA	0.084	0.910	NA	NA	0.013	1.000
NM_003705	SLC25A12	NA	NA	NA	NA	-0.534	0.158	NA	NA	0.069	1.000
NM_001160210	SLC25A13	NA	NA	NA	NA	0.325	0.464	NA	NA	-0.175	0.907
NM_014252	SLC25A15	NA	NA	NA	NA	0.568	0.340	NA	NA	0.593	0.582
NM_001303484	SLC25A18	NA	NA	NA	NA	ND	ND	NA	NA	ND	ND
NM_000387	SLC25A20	NA	NA	NA	NA	0.598	0.090	NA	NA	0.570	0.140
NM_001191061	SLC25A22	NA	NA	NA	NA	-0.209	0.682	NA	NA	0.323	0.614
NM_001039355	SLC25A29	NA	NA	NA	NA	-0.205	0.755	NA	NA	0.728	0.061
NM_178526	SLC25A42	NA	NA	NA	NA	-0.579	0.349	NA	NA	0.086	1.000

	NM_198580	SLC27A1	NA	NA	NA	NA	0.625	0.098	NA	NA	0.427	0.677
	NM_003850	SUCLA2	NA	NA	NA	NA	-0.054	0.953	NA	NA	-0.030	1.000
	NM_003849	SUCLG1	NA	NA	NA	NA	-0.052	0.956	NA	NA	-0.013	1.000
	NM_001177599	SUCLG2	NA	NA	NA	NA	-0.108	0.890	NA	NA	-0.281	0.766
	NM_003355	UCP2	NA	NA	NA	NA	-0.047	0.968	NA	NA	0.333	0.596
G25+Olea	NM_001098	ACO2	NA	NA	NA	NA	0.112	0.850	0.824	0.001	-0.112	0.927
	NM_004077	CS	NA	NA	NA	NA	-0.019	0.998	-0.078	0.819	-0.241	0.713
	NM_000143	FH	NA	NA	NA	NA	-0.195	0.702	-0.034	0.933	-0.233	0.740
	NM_012084	GLUD2	NA	NA	NA	NA	-0.141	0.945	-0.159	0.878	0.832	0.595
	NM_002080	GOT2	NA	NA	NA	NA	0.004	1.000	-0.165	0.585	-0.286	0.595
	NM_002168	IDH2	NA	NA	NA	NA	0.435	0.180	0.304	0.319	-0.141	0.885
	NM_005530	IDH3A	NA	NA	NA	NA	0.218	0.630	0.156	0.619	0.010	1.000
	NM_174855	IDH3B	NA	NA	NA	NA	-0.242	0.591	0.522	0.057	-0.160	0.862
	NM_174869	IDH3G	NA	NA	NA	NA	-0.218	0.644	-0.287	0.345	-0.236	0.739
	NM_001282404	MDH2	NA	NA	NA	NA	0.056	0.942	0.511	0.043	-0.256	0.645
	NM_016098	MPC1	NA	NA	NA	NA	-0.440	0.212	0.446	0.102	-0.157	0.866
	NM_001143674	MPC2	NA	NA	NA	NA	-0.070	0.921	0.600	0.017	0.122	0.900
	NM_001165036	OGDH	NA	NA	NA	NA	0.093	0.885	0.745	0.002	-0.062	0.968
	NM_000920	PC	NA	NA	NA	NA	-0.059	0.954	-0.863	0.001	-0.631	0.138
	NM_004563	PCK2	NA	NA	NA	NA	-0.183	0.748	0.065	0.862	-0.059	0.978
	NM_001173456	PDHA1	NA	NA	NA	NA	0.099	0.873	1.101	0.000	0.085	0.951
	NM_001173468	PDHB	NA	NA	NA	NA	-0.114	0.843	0.101	0.769	-0.086	0.946
	NM_003477	PDHX	NA	NA	NA	NA	-0.206	0.675	-0.434	0.116	-0.049	0.983
	NM_002610	PDK1	NA	NA	NA	NA	-0.281	0.484	-0.237	0.503	0.504	0.172
	NM_001199898	PDK2	NA	NA	NA	NA	0.228	0.649	0.082	0.857	-0.118	0.931
	NM_001142386	PDK3	NA	NA	NA	NA	-0.271	0.505	-0.300	0.272	0.008	1.000
	NM_002612	PDK4	NA	NA	NA	NA	-0.136	0.796	0.105	0.737	-0.083	0.947
	NM_001294332	SDHA	NA	NA	NA	NA	0.528	0.084	0.265	0.352	-0.149	0.872

	NM_003000	SDHB	NA	NA	NA	NA	-0.105	0.861	0.428	0.113	-0.067	0.962
	NM_003001	SDHC	NA	NA	NA	NA	-0.114	0.847	0.247	0.421	-0.036	0.994
	NM_001276503	SDHD	NA	NA	NA	NA	-0.033	0.980	0.340	0.238	0.181	0.815
	NM_005984	SLC25A1	NA	NA	NA	NA	0.275	0.497	-0.024	0.959	0.701	0.019
	NM_012140	SLC25A10	NA	NA	NA	NA	0.795	0.010	1.183	0.000	0.034	1.000
	NM_001165418	SLC25A11	NA	NA	NA	NA	0.057	0.945	0.131	0.685	-0.270	0.634
	NM_003705	SLC25A12	NA	NA	NA	NA	-0.453	0.235	0.164	0.614	0.006	1.000
	NM_001160210	SLC25A13	NA	NA	NA	NA	0.325	0.422	0.177	0.585	-0.420	0.296
	NM_014252	SLC25A15	NA	NA	NA	NA	0.646	0.214	-0.138	0.764	0.234	0.921
	NM_001303484	SLC25A18	NA	NA	NA	NA	ND	ND	-0.515	0.276	ND	ND
	NM_000387	SLC25A20	NA	NA	NA	NA	0.368	0.380	1.484	0.000	0.415	0.350
	NM_001191061	SLC25A22	NA	NA	NA	NA	-0.125	0.825	1.618	0.000	0.093	0.943
	NM_001039355	SLC25A29	NA	NA	NA	NA	0.199	0.740	-0.131	0.686	0.558	0.194
	NM_178526	SLC25A42	NA	NA	NA	NA	-0.383	0.567	0.508	0.065	-0.259	0.824
	NM_198580	SLC27A1	NA	NA	NA	NA	0.724	0.031	0.386	0.193	0.450	0.536
	NM_003850	SUCLA2	NA	NA	NA	NA	-0.114	0.846	-0.512	0.060	-0.091	0.942
	NM_003849	SUCLG1	NA	NA	NA	NA	-0.031	0.982	-0.023	0.961	0.053	0.978
	NM_001177599	SUCLG2	NA	NA	NA	NA	-0.028	0.990	-0.498	0.099	-0.212	0.804
	NM_003355	UCP2	NA	NA	NA	NA	0.215	0.696	0.306	0.370	0.005	1.000
G25+Palm	NM_001098	ACO2	NA	NA	NA	NA	-0.138	0.778	NA	NA	0.031	1.000
	NM_004077	CS	NA	NA	NA	NA	0.055	0.935	NA	NA	-0.146	0.870
	NM_000143	FH	NA	NA	NA	NA	-0.211	0.631	NA	NA	-0.119	0.907
	NM_012084	GLUD2	NA	NA	NA	NA	0.210	0.902	NA	NA	0.466	0.868
	NM_002080	GOT2	NA	NA	NA	NA	-0.105	0.844	NA	NA	0.042	0.989
	NM_002168	IDH2	NA	NA	NA	NA	0.256	0.482	NA	NA	-0.320	0.524
	NM_005530	IDH3A	NA	NA	NA	NA	0.075	0.901	NA	NA	0.011	1.000
	NM_174855	IDH3B	NA	NA	NA	NA	-0.006	1.000	NA	NA	-0.412	0.324
	NM_174869	IDH3G	NA	NA	NA	NA	-0.231	0.572	NA	NA	0.017	1.000

	NM_001282404	MDH2	NA	NA	NA	NA	-0.117	0.816	NA	NA	-0.201	0.758
	NM_016098	MPC1	NA	NA	NA	NA	-0.111	0.841	NA	NA	0.439	0.270
	NM_001143674	MPC2	NA	NA	NA	NA	-0.211	0.598	NA	NA	0.327	0.467
	NM_001165036	OGDH	NA	NA	NA	NA	-0.111	0.837	NA	NA	0.068	0.971
	NM_000920	PC	NA	NA	NA	NA	-0.268	0.532	NA	NA	-0.216	0.806
	NM_004563	PCK2	NA	NA	NA	NA	-0.228	0.616	NA	NA	-0.051	0.990
	NM_001173456	PDHA1	NA	NA	NA	NA	0.107	0.843	NA	NA	0.063	0.976
	NM_001173468	PDHB	NA	NA	NA	NA	-0.292	0.406	NA	NA	-0.121	0.893
	NM_003477	PDHX	NA	NA	NA	NA	-0.215	0.616	NA	NA	-0.086	0.952
	NM_002610	PDK1	NA	NA	NA	NA	0.143	0.761	NA	NA	0.014	1.000
	NM_001199898	PDK2	NA	NA	NA	NA	-0.070	0.924	NA	NA	-0.117	0.922
	NM_001142386	PDK3	NA	NA	NA	NA	-0.320	0.345	NA	NA	0.113	0.905
	NM_002612	PDK4	NA	NA	NA	NA	0.338	0.296	NA	NA	1.876	0.000
	NM_001294332	SDHA	NA	NA	NA	NA	0.241	0.525	NA	NA	-0.084	0.952
	NM_003000	SDHB	NA	NA	NA	NA	-0.084	0.886	NA	NA	0.032	1.000
	NM_003001	SDHC	NA	NA	NA	NA	-0.365	0.277	NA	NA	-0.257	0.660
	NM_001276503	SDHD	NA	NA	NA	NA	-0.001	1.000	NA	NA	0.186	0.795
	NM_005984	SLC25A1	NA	NA	NA	NA	0.094	0.864	NA	NA	0.468	0.215
	NM_012140	SLC25A10	NA	NA	NA	NA	0.557	0.090	NA	NA	0.166	0.862
	NM_001165418	SLC25A11	NA	NA	NA	NA	-0.184	0.675	NA	NA	0.011	1.000
	NM_003705	SLC25A12	NA	NA	NA	NA	-0.572	0.077	NA	NA	-0.022	1.000
	NM_001160210	SLC25A13	NA	NA	NA	NA	0.262	0.511	NA	NA	-0.388	0.368
	NM_014252	SLC25A15	NA	NA	NA	NA	0.814	0.048	NA	NA	0.857	0.154
	NM_001303484	SLC25A18	NA	NA	NA	NA	ND	ND	NA	NA	ND	ND
	NM_000387	SLC25A20	NA	NA	NA	NA	0.547	0.090	NA	NA	0.503	0.196
	NM_001191061	SLC25A22	NA	NA	NA	NA	-0.337	0.323	NA	NA	0.320	0.516
	NM_001039355	SLC25A29	NA	NA	NA	NA	0.185	0.727	NA	NA	0.773	0.030
	NM_178526	SLC25A42	NA	NA	NA	NA	-0.191	0.795	NA	NA	-0.189	0.882

NM_198580	SLC27A1	NA	NA	NA	NA	0.376	0.354	NA	NA	0.457	0.507
NM_003850	SUCLA2	NA	NA	NA	NA	-0.085	0.883	NA	NA	-0.107	0.916
NM_003849	SUCLG1	NA	NA	NA	NA	0.022	0.983	NA	NA	0.155	0.851
NM_001177599	SUCLG2	NA	NA	NA	NA	0.139	0.800	NA	NA	-0.143	0.896
NM_003355	UCP2	NA	NA	NA	NA	-0.751	0.017	NA	NA	0.594	0.068

Supplementary Table S3. Quantitative data related to the transcriptomic profiles of the electron transport chain machinery and related mitochondrial carriers in human islets upon metabolic stress.NA: Not applicable (condition not tested); ND: not detected.

			**Donor #1**		**Donor #2**		**Donor #3**		**Donor #4**		**Donor #5**	
	Accession (NCBI Ref. Sequence)	Gene symbol	**Log$_2$ FC**	**Adjusted *p***	**Log$_2$ FC**	**Adjusted *p***	**Log$_2$ FC**	**Adjusted *p***	**Log$_2$ FC**	**Adjusted *p***	**Log$_2$ FC**	**Adjusted *p***
Olea	NM_001001937	ATP5A1	-0.242	0.489	0.037	0.972	NA	NA	-0.265	0.491	NA	NA
	NM_001686	ATP5B	0.177	0.637	0.139	0.798	NA	NA	0.099	0.852	NA	NA
	NM_005174	ATP5C1	-0.089	0.860	0.270	0.560	NA	NA	-0.252	0.544	NA	NA
	NM_001687	ATP5D	0.892	0.075	0.670	0.120	NA	NA	-0.118	0.837	NA	NA
	NM_001688	ATP5F1	-0.331	0.371	-0.088	0.899	NA	NA	-0.239	0.575	NA	NA
	NM_005175	ATP5G1	0.321	0.642	0.877	0.057	NA	NA	0.152	0.801	NA	NA
	NM_001002031	ATP5G2	0.762	0.018	0.257	0.579	NA	NA	0.214	0.613	NA	NA
	NM_001689	ATP5G3	-0.167	0.715	0.111	0.863	NA	NA	0.162	0.728	NA	NA
	NM_006356	ATP5H	-0.113	0.844	0.249	0.641	NA	NA	0.309	0.455	NA	NA
	NM_007100	ATP5I	0.167	0.847	0.489	0.345	NA	NA	0.400	0.402	NA	NA
	NM_004889	ATP5J2	-0.246	0.584	0.449	0.305	NA	NA	0.575	0.091	NA	NA
	NM_006476	ATP5L	-0.219	0.573	0.053	0.948	NA	NA	-0.010	1.000	NA	NA
	NM_001697	ATP5O	-0.473	0.180	-0.005	1.000	NA	NA	-0.258	0.542	NA	NA
	NM_001003805	ATP5S	-0.142	0.924	-0.043	0.992	NA	NA	-0.292	0.575	NA	NA
	NM_178190	ATPIF1	-0.312	0.489	0.248	0.630	NA	NA	0.188	0.680	NA	NA
	NM_001162862	COX11	-0.600	0.302	-0.393	0.538	NA	NA	-0.285	0.498	NA	NA
	NM_078470	COX15	0.016	1.000	-0.393	0.387	NA	NA	-0.042	0.958	NA	NA
	NM_005694	COX17	-0.091	0.919	0.347	0.495	NA	NA	0.083	0.899	NA	NA
	NM_001861	COX4I1	-0.272	0.447	0.374	0.360	NA	NA	0.270	0.484	NA	NA
	NM_004255	COX5A	0.183	0.747	-0.006	1.000	NA	NA	-0.128	0.819	NA	NA
	NM_001862	COX5B	1.358	0.000	0.792	0.038	NA	NA	0.277	0.495	NA	NA
	NM_004373	COX6A1	0.274	0.627	0.369	0.465	NA	NA	0.027	0.983	NA	NA

	NM_001863	COX6B1	-0.074	0.910	0.479	0.244	NA	NA	0.035	0.971	NA	NA
	NM_004374	COX6C	-0.431	0.246	0.207	0.692	NA	NA	0.132	0.806	NA	NA
	NM_001864	COX7A1	0.668	0.638	-0.131	0.935	NA	NA	0.171	0.815	NA	NA
	NM_001865	COX7A2	-0.541	0.107	0.408	0.341	NA	NA	0.098	0.862	NA	NA
	NM_004718	COX7A2L	-0.176	0.666	0.085	0.899	NA	NA	-0.016	0.997	NA	NA
	NM_001866	COX7B	-0.555	0.122	0.255	0.617	NA	NA	0.343	0.389	NA	NA
	NM_001867	COX7C	-0.120	0.796	0.336	0.447	NA	NA	0.145	0.761	NA	NA
	NM_004074	COX8A	1.761	0.000	0.571	0.129	NA	NA	0.274	0.477	NA	NA
	NM_004541	NDUFA1	-0.047	0.982	0.587	0.158	NA	NA	0.090	0.883	NA	NA
	NM_004544	NDUFA10	0.139	0.782	0.204	0.692	NA	NA	-0.142	0.777	NA	NA
	NM_001258338	NDUFA12	-0.268	0.571	0.301	0.557	NA	NA	-0.170	0.733	NA	NA
	NM_002488	NDUFA2	0.151	0.809	0.691	0.119	NA	NA	0.163	0.748	NA	NA
	NM_004542	NDUFA3	0.971	0.092	0.390	0.459	NA	NA	-0.067	0.940	NA	NA
	NM_002489	NDUFA4	-0.481	0.253	0.229	0.703	NA	NA	0.391	0.309	NA	NA
	NM_001291304	NDUFA5	-0.816	0.005	0.117	0.856	NA	NA	-0.375	0.300	NA	NA
	NM_002490	NDUFA6	-0.119	0.808	0.435	0.328	NA	NA	0.032	0.976	NA	NA
	NM_005001	NDUFA7	0.834	0.140	0.578	0.180	NA	NA	0.259	0.597	NA	NA
	NM_014222	NDUFA8	-0.323	0.518	0.378	0.439	NA	NA	0.102	0.865	NA	NA
	NM_005002	NDUFA9	-0.232	0.594	0.406	0.371	NA	NA	-0.242	0.578	NA	NA
	NM_005003	NDUFAB1	-0.395	0.374	0.550	0.203	NA	NA	0.156	0.761	NA	NA
	NM_004545	NDUFB1	-0.503	0.303	0.107	0.902	NA	NA	0.258	0.658	NA	NA
	NM_004548	NDUFB10	0.496	0.258	0.580	0.165	NA	NA	-0.023	0.990	NA	NA
	NM_004546	NDUFB2	0.467	0.301	0.515	0.245	NA	NA	0.196	0.678	NA	NA
	NM_002491	NDUFB3	-0.545	0.204	-0.177	0.792	NA	NA	-0.057	0.956	NA	NA
	NM_004547	NDUFB4	0.311	0.426	0.284	0.542	NA	NA	-0.002	1.000	NA	NA
	NM_001199958	NDUFB5	-0.497	0.145	0.601	0.131	NA	NA	-0.063	0.927	NA	NA
	NM_002493	NDUFB6	0.372	0.429	0.488	0.279	NA	NA	-0.025	0.992	NA	NA
	NM_004146	NDUFB7	1.200	0.002	0.535	0.191	NA	NA	0.347	0.352	NA	NA

NM_005004	NDUFB8	0.140	0.792	0.452	0.288	NA	NA	-0.059	0.929	NA	NA
NM_005005	NDUFB9	-0.061	0.927	0.309	0.506	NA	NA	-0.071	0.913	NA	NA
NM_001184990	NDUFC1	0.475	0.321	0.476	0.314	NA	NA	0.109	0.870	NA	NA
NM_001204055	NDUFC2	-0.754	0.026	-0.058	0.949	NA	NA	0.196	0.685	NA	NA
NM_001199981	NDUFS1	-0.299	0.436	-0.233	0.653	NA	NA	-0.395	0.257	NA	NA
NM_004550	NDUFS2	-0.024	1.000	0.030	0.993	NA	NA	0.098	0.862	NA	NA
NM_004551	NDUFS3	-0.038	0.996	0.137	0.836	NA	NA	-0.029	0.987	NA	NA
NM_002495	NDUFS4	-0.295	0.570	0.187	0.782	NA	NA	-0.190	0.724	NA	NA
NM_004552	NDUFS5	-0.540	0.099	0.283	0.541	NA	NA	0.165	0.743	NA	NA
NM_004553	NDUFS6	0.599	0.196	0.447	0.348	NA	NA	-0.244	0.569	NA	NA
NM_024407	NDUFS7	1.302	0.003	0.941	0.021	NA	NA	0.271	0.509	NA	NA
NM_002496	NDUFS8	1.240	0.002	0.546	0.206	NA	NA	0.205	0.660	NA	NA
NM_001166102	NDUFV1	0.441	0.262	0.633	0.105	NA	NA	0.082	0.889	NA	NA
NM_021074	NDUFV2	-0.300	0.485	0.064	0.941	NA	NA	0.051	0.947	NA	NA
NM_021075	NDUFV3	0.571	0.197	0.084	0.936	NA	NA	0.089	0.892	NA	NA
NM_004589	SCO1	0.285	0.589	0.117	0.876	NA	NA	-0.001	1.000	NA	NA
NM_001294332	SDHA	-0.219	0.627	0.026	0.995	NA	NA	-0.189	0.679	NA	NA
NM_003000	SDHB	0.140	0.801	0.444	0.308	NA	NA	0.011	1.000	NA	NA
NM_003001	SDHC	-0.148	0.795	-0.231	0.704	NA	NA	0.267	0.538	NA	NA
NM_001276503	SDHD	-0.169	0.728	0.165	0.793	NA	NA	-0.190	0.697	NA	NA
NM_001282198	SLC25A14	-0.783	0.090	0.136	0.875	NA	NA	-0.031	0.986	NA	NA
NM_001204052	SLC25A27	-0.610	0.137	-0.418	0.483	NA	NA	0.348	0.400	NA	NA
NM_002635	SLC25A3	-0.153	0.700	0.042	0.966	NA	NA	-0.099	0.854	NA	NA
NM_001151	SLC25A4	-0.020	1.000	0.233	0.642	NA	NA	-0.107	0.842	NA	NA
NM_001152	SLC25A5	-0.294	0.403	0.225	0.639	NA	NA	-0.232	0.567	NA	NA
NM_003172	SURF1	-0.212	0.731	0.361	0.491	NA	NA	0.221	0.618	NA	NA
NM_003355	UCP2	0.167	0.829	0.252	0.685	NA	NA	0.257	0.619	NA	NA
NM_013387	UQCR10	0.601	0.102	0.391	0.382	NA	NA	0.287	0.480	NA	NA

	NM_006830	UQCR11	0.875	0.013	0.476	0.248	NA	NA	0.191	0.672	NA	NA
	NM_006294	UQCRB	-0.519	0.102	0.090	0.891	NA	NA	-0.326	0.383	NA	NA
	NM_003365	UQCRC1	0.443	0.232	0.284	0.545	NA	NA	0.090	0.878	NA	NA
	NM_003366	UQCRC2	-0.387	0.271	0.151	0.792	NA	NA	-0.448	0.184	NA	NA
	NM_006003	UQCRFS1	-0.488	0.194	0.183	0.751	NA	NA	-0.034	0.972	NA	NA
	NM_001297566	UQCRH	0.177	0.729	0.177	0.762	NA	NA	-0.082	0.900	NA	NA
	NM_014402	UQCRQ	-0.116	0.806	0.287	0.534	NA	NA	0.123	0.819	NA	NA
Palm	NM_001001937	ATP5A1	-0.088	0.870	0.006	1.000	NA	NA	NA	NA	NA	NA
	NM_001686	ATP5B	0.272	0.449	0.039	1.000	NA	NA	NA	NA	NA	NA
	NM_005174	ATP5C1	0.254	0.522	-0.015	1.000	NA	NA	NA	NA	NA	NA
	NM_001687	ATP5D	1.104	0.018	0.512	1.000	NA	NA	NA	NA	NA	NA
	NM_001688	ATP5F1	0.095	0.880	-0.010	1.000	NA	NA	NA	NA	NA	NA
	NM_005175	ATP5G1	1.028	0.028	0.579	1.000	NA	NA	NA	NA	NA	NA
	NM_001002031	ATP5G2	0.850	0.009	0.196	1.000	NA	NA	NA	NA	NA	NA
	NM_001689	ATP5G3	0.357	0.359	0.030	1.000	NA	NA	NA	NA	NA	NA
	NM_006356	ATP5H	0.568	0.135	0.123	1.000	NA	NA	NA	NA	NA	NA
	NM_007100	ATP5I	0.094	0.973	0.681	0.900	NA	NA	NA	NA	NA	NA
	NM_004889	ATP5J2	0.016	1.000	0.194	1.000	NA	NA	NA	NA	NA	NA
	NM_006476	ATP5L	0.128	0.797	-0.029	1.000	NA	NA	NA	NA	NA	NA
	NM_001697	ATP5O	0.053	0.956	-0.196	1.000	NA	NA	NA	NA	NA	NA
	NM_001003805	ATP5S	-0.402	0.557	-0.348	1.000	NA	NA	NA	NA	NA	NA
	NM_178190	ATPIF1	0.355	0.424	-0.127	1.000	NA	NA	NA	NA	NA	NA
	NM_001162862	COX11	0.063	0.983	0.242	1.000	NA	NA	NA	NA	NA	NA
	NM_078470	COX15	0.226	0.628	-0.275	1.000	NA	NA	NA	NA	NA	NA
	NM_005694	COX17	0.279	0.617	0.214	1.000	NA	NA	NA	NA	NA	NA
	NM_001861	COX4I1	-0.127	0.799	0.067	1.000	NA	NA	NA	NA	NA	NA
	NM_004255	COX5A	-0.038	0.999	-0.067	1.000	NA	NA	NA	NA	NA	NA
	NM_001862	COX5B	1.481	0.000	0.654	0.751	NA	NA	NA	NA	NA	NA

	NM_004373	COX6A1	0.589	0.194	0.412	1.000	NA	NA	NA	NA	NA	NA
	NM_001863	COX6B1	0.177	0.724	0.326	1.000	NA	NA	NA	NA	NA	NA
	NM_004374	COX6C	0.169	0.734	-0.135	1.000	NA	NA	NA	NA	NA	NA
	NM_001864	COX7A1	0.141	1.000	-0.217	1.000	NA	NA	NA	NA	NA	NA
	NM_001865	COX7A2	-0.083	0.897	0.401	1.000	NA	NA	NA	NA	NA	NA
	NM_004718	COX7A2L	0.118	0.819	-0.005	1.000	NA	NA	NA	NA	NA	NA
	NM_001866	COX7B	-0.242	0.603	0.017	1.000	NA	NA	NA	NA	NA	NA
	NM_001867	COX7C	0.307	0.418	0.138	1.000	NA	NA	NA	NA	NA	NA
	NM_004074	COX8A	1.786	0.000	0.230	1.000	NA	NA	NA	NA	NA	NA
	NM_004541	NDUFA1	0.129	0.861	0.628	0.790	NA	NA	NA	NA	NA	NA
	NM_004544	NDUFA10	0.359	0.363	0.063	1.000	NA	NA	NA	NA	NA	NA
	NM_001258338	NDUFA12	0.208	0.699	0.126	1.000	NA	NA	NA	NA	NA	NA
	NM_002488	NDUFA2	-0.053	0.972	0.298	1.000	NA	NA	NA	NA	NA	NA
	NM_004542	NDUFA3	0.364	0.756	0.229	1.000	NA	NA	NA	NA	NA	NA
	NM_002489	NDUFA4	0.014	1.000	-0.547	1.000	NA	NA	NA	NA	NA	NA
	NM_001291304	NDUFA5	-0.067	0.928	0.112	1.000	NA	NA	NA	NA	NA	NA
	NM_002490	NDUFA6	0.292	0.482	0.221	1.000	NA	NA	NA	NA	NA	NA
	NM_005001	NDUFA7	1.487	0.001	0.255	1.000	NA	NA	NA	NA	NA	NA
	NM_014222	NDUFA8	0.218	0.705	0.411	1.000	NA	NA	NA	NA	NA	NA
	NM_005002	NDUFA9	-0.054	0.955	-0.120	1.000	NA	NA	NA	NA	NA	NA
	NM_005003	NDUFAB1	0.157	0.806	0.366	1.000	NA	NA	NA	NA	NA	NA
	NM_004545	NDUFB1	0.090	0.944	0.283	1.000	NA	NA	NA	NA	NA	NA
	NM_004548	NDUFB10	0.585	0.166	0.273	1.000	NA	NA	NA	NA	NA	NA
	NM_004546	NDUFB2	0.789	0.045	0.175	1.000	NA	NA	NA	NA	NA	NA
	NM_002491	NDUFB3	-0.477	0.302	-0.071	1.000	NA	NA	NA	NA	NA	NA
	NM_004547	NDUFB4	0.485	0.179	0.307	1.000	NA	NA	NA	NA	NA	NA
	NM_001199958	NDUFB5	0.073	0.918	0.329	1.000	NA	NA	NA	NA	NA	NA
	NM_002493	NDUFB6	0.806	0.040	0.105	1.000	NA	NA	NA	NA	NA	NA

	NM_004146	NDUFB7	1.325	0.001	-0.025	1.000	NA	NA	NA	NA	NA	NA
	NM_005004	NDUFB8	0.314	0.486	0.456	1.000	NA	NA	NA	NA	NA	NA
	NM_005005	NDUFB9	0.153	0.768	0.404	1.000	NA	NA	NA	NA	NA	NA
	NM_001184990	NDUFC1	0.378	0.497	0.209	1.000	NA	NA	NA	NA	NA	NA
	NM_001204055	NDUFC2	-0.089	0.900	-0.241	1.000	NA	NA	NA	NA	NA	NA
	NM_001199981	NDUFS1	-0.075	0.923	0.172	1.000	NA	NA	NA	NA	NA	NA
	NM_004550	NDUFS2	0.153	0.785	0.203	1.000	NA	NA	NA	NA	NA	NA
	NM_004551	NDUFS3	0.434	0.318	-0.207	1.000	NA	NA	NA	NA	NA	NA
	NM_002495	NDUFS4	-0.066	0.967	0.045	1.000	NA	NA	NA	NA	NA	NA
	NM_004552	NDUFS5	-0.257	0.527	0.016	1.000	NA	NA	NA	NA	NA	NA
	NM_004553	NDUFS6	0.868	0.044	0.193	1.000	NA	NA	NA	NA	NA	NA
	NM_024407	NDUFS7	0.922	0.074	0.484	1.000	NA	NA	NA	NA	NA	NA
	NM_002496	NDUFS8	1.242	0.002	0.213	1.000	NA	NA	NA	NA	NA	NA
	NM_001166102	NDUFV1	0.461	0.253	0.270	1.000	NA	NA	NA	NA	NA	NA
	NM_021074	NDUFV2	-0.079	0.918	0.059	1.000	NA	NA	NA	NA	NA	NA
	NM_021075	NDUFV3	0.433	0.398	0.078	1.000	NA	NA	NA	NA	NA	NA
	NM_004589	SCO1	0.390	0.435	-0.071	1.000	NA	NA	NA	NA	NA	NA
	NM_001294332	SDHA	-0.225	0.663	0.043	1.000	NA	NA	NA	NA	NA	NA
	NM_003000	SDHB	0.195	0.724	0.136	1.000	NA	NA	NA	NA	NA	NA
	NM_003001	SDHC	-0.371	0.466	-0.261	1.000	NA	NA	NA	NA	NA	NA
	NM_001276503	SDHD	0.060	0.959	0.088	1.000	NA	NA	NA	NA	NA	NA
	NM_001282198	SLC25A14	0.131	0.878	0.421	1.000	NA	NA	NA	NA	NA	NA
	NM_001204052	SLC25A27	-0.266	0.600	-0.666	1.000	NA	NA	NA	NA	NA	NA
	NM_002635	SLC25A3	0.111	0.823	-0.075	1.000	NA	NA	NA	NA	NA	NA
	NM_001151	SLC25A4	0.292	0.481	0.085	1.000	NA	NA	NA	NA	NA	NA
	NM_001152	SLC25A5	-0.085	0.886	0.179	1.000	NA	NA	NA	NA	NA	NA
	NM_003172	SURF1	0.158	0.819	0.004	1.000	NA	NA	NA	NA	NA	NA
	NM_003355	UCP2	0.506	0.413	-0.048	1.000	NA	NA	NA	NA	NA	NA

	NM_013387	UQCR10	0.680	0.060	0.166	1.000	NA	NA	NA	NA	NA	NA
	NM_006830	UQCR11	1.069	0.002	0.150	1.000	NA	NA	NA	NA	NA	NA
	NM_006294	UQCRB	-0.200	0.632	0.053	1.000	NA	NA	NA	NA	NA	NA
	NM_003365	UQCRC1	0.675	0.047	0.190	1.000	NA	NA	NA	NA	NA	NA
	NM_003366	UQCRC2	-0.023	1.000	0.101	1.000	NA	NA	NA	NA	NA	NA
	NM_006003	UQCRFS1	-0.210	0.667	0.018	1.000	NA	NA	NA	NA	NA	NA
	NM_001297566	UQCRH	0.252	0.640	0.090	1.000	NA	NA	NA	NA	NA	NA
	NM_014402	UQCRQ	0.510	0.127	0.101	1.000	NA	NA	NA	NA	NA	NA
G25	NM_001001937	ATP5A1	-0.127	0.727	0.225	0.556	-0.343	0.359	-0.307	0.280	NA	NA
	NM_001686	ATP5B	0.257	0.406	0.392	0.236	-0.098	0.885	0.860	0.000	NA	NA
	NM_005174	ATP5C1	0.039	0.950	0.400	0.258	-0.160	0.768	-0.096	0.796	NA	NA
	NM_001687	ATP5D	1.330	0.002	0.909	0.013	-0.088	0.909	0.011	1.000	NA	NA
	NM_001688	ATP5F1	-0.038	0.964	0.131	0.800	-0.323	0.418	0.544	0.044	NA	NA
	NM_005175	ATP5G1	1.146	0.008	1.070	0.009	-0.185	0.736	1.037	0.000	NA	NA
	NM_001002031	ATP5G2	1.219	0.000	0.227	0.575	-0.453	0.183	-1.047	0.000	NA	NA
	NM_001689	ATP5G3	0.322	0.347	0.316	0.412	-0.326	0.410	0.393	0.159	NA	NA
	NM_006356	ATP5H	0.569	0.103	0.559	0.131	-0.371	0.328	-0.156	0.670	NA	NA
	NM_007100	ATP5I	0.657	0.177	0.664	0.140	-0.298	0.481	0.617	0.060	NA	NA
	NM_004889	ATP5J2	0.042	0.971	0.786	0.018	-0.250	0.577	0.854	0.001	NA	NA
	NM_006476	ATP5L	0.044	0.940	0.297	0.428	-0.499	0.120	-0.396	0.156	NA	NA
	NM_001697	ATP5O	-0.322	0.356	0.177	0.714	-0.013	1.000	-0.024	0.969	NA	NA
	NM_001003805	ATP5S	-0.157	0.852	-0.355	0.682	-0.409	0.359	-0.339	0.359	NA	NA
	NM_178190	ATPIF1	0.432	0.247	0.599	0.092	-0.157	0.776	0.612	0.021	NA	NA
	NM_001162862	COX11	-0.604	0.251	-0.022	1.000	-0.217	0.696	0.604	0.026	NA	NA
	NM_078470	COX15	-0.236	0.568	-0.443	0.272	0.045	0.971	0.145	0.668	NA	NA
	NM_005694	COX17	0.560	0.163	0.549	0.164	-0.368	0.327	-1.080	0.000	NA	NA
	NM_001861	COX4I1	0.185	0.597	0.587	0.056	-0.122	0.843	-0.302	0.295	NA	NA
	NM_004255	COX5A	0.217	0.644	0.041	0.977	-0.244	0.604	0.029	0.960	NA	NA

	NM_001862	COX5B	1.615	0.000	0.945	0.004	-0.449	0.188	0.144	0.677	NA	NA
	NM_004373	COX6A1	0.446	0.319	0.633	0.102	-0.419	0.242	0.410	0.150	NA	NA
	NM_001863	COX6B1	0.275	0.450	0.842	0.007	-0.415	0.253	-0.033	0.948	NA	NA
	NM_004374	COX6C	-0.102	0.835	0.471	0.190	-0.683	0.017	-0.919	0.001	NA	NA
	NM_001864	COX7A1	1.208	0.201	0.308	0.765	-0.018	1.000	-0.922	0.016	NA	NA
	NM_001865	COX7A2	-0.079	0.871	0.987	0.001	-0.347	0.355	-0.765	0.003	NA	NA
	NM_004718	COX7A2L	0.097	0.816	0.148	0.745	-0.061	0.943	0.469	0.080	NA	NA
	NM_001866	COX7B	-0.076	0.882	0.425	0.276	-0.600	0.050	-0.551	0.064	NA	NA
	NM_001867	COX7C	0.231	0.507	0.527	0.110	-0.563	0.070	-0.251	0.406	NA	NA
	NM_004074	COX8A	1.755	0.000	0.663	0.033	-0.605	0.043	-0.116	0.737	NA	NA
	NM_004541	NDUFA1	0.351	0.374	1.060	0.001	-0.498	0.131	0.081	0.843	NA	NA
	NM_004544	NDUFA10	0.129	0.772	0.207	0.648	-0.022	0.996	0.650	0.012	NA	NA
	NM_001258338	NDUFA12	0.058	0.933	0.557	0.147	-0.081	0.917	-0.035	0.946	NA	NA
	NM_002488	NDUFA2	0.290	0.570	1.013	0.006	-0.368	0.344	0.228	0.486	NA	NA
	NM_004542	NDUFA3	1.375	0.006	0.236	0.665	-0.414	0.269	0.103	0.826	NA	NA
	NM_002489	NDUFA4	-0.161	0.734	0.249	0.630	-0.260	0.558	0.446	0.137	NA	NA
	NM_001291304	NDUFA5	-0.462	0.130	0.197	0.671	-0.090	0.900	0.406	0.148	NA	NA
	NM_002490	NDUFA6	0.029	0.975	0.417	0.297	0.020	0.998	1.198	0.000	NA	NA
	NM_005001	NDUFA7	1.299	0.005	0.677	0.072	-0.772	0.007	-0.309	0.395	NA	NA
	NM_014222	NDUFA8	-0.021	1.000	0.380	0.403	-0.068	0.939	0.568	0.037	NA	NA
	NM_005002	NDUFA9	-0.378	0.293	0.204	0.687	-0.158	0.774	0.431	0.130	NA	NA
	NM_005003	NDUFAB1	-0.143	0.809	0.712	0.051	-0.235	0.611	0.315	0.300	NA	NA
	NM_004545	NDUFB1	0.205	0.705	0.798	0.053	-0.737	0.014	-0.991	0.007	NA	NA
	NM_004548	NDUFB10	0.651	0.092	0.811	0.018	-0.188	0.715	0.676	0.010	NA	NA
	NM_004546	NDUFB2	0.907	0.011	0.811	0.022	-0.377	0.303	-0.231	0.490	NA	NA
	NM_002491	NDUFB3	0.042	0.965	0.332	0.509	-0.572	0.078	0.452	0.169	NA	NA
	NM_004547	NDUFB4	0.303	0.398	0.450	0.207	-0.381	0.303	0.550	0.038	NA	NA
	NM_001199958	NDUFB5	-0.279	0.425	0.751	0.025	-0.328	0.410	-0.037	0.937	NA	NA

	NM_002493	NDUFB6	0.572	0.146	0.482	0.256	-0.341	0.381	0.354	0.252	NA	NA
	NM_004146	NDUFB7	1.348	0.000	0.436	0.263	-0.350	0.361	0.419	0.142	NA	NA
	NM_005004	NDUFB8	0.347	0.362	0.625	0.068	-0.306	0.442	-0.275	0.354	NA	NA
	NM_005005	NDUFB9	0.268	0.453	0.642	0.048	-0.321	0.418	1.132	0.000	NA	NA
	NM_001184990	NDUFC1	0.341	0.476	0.387	0.409	-0.235	0.612	0.305	0.366	NA	NA
	NM_001204055	NDUFC2	-0.279	0.453	0.287	0.514	-0.189	0.714	-0.375	0.230	NA	NA
	NM_001199981	NDUFS1	-0.232	0.536	-0.407	0.310	-0.216	0.656	0.172	0.599	NA	NA
	NM_004550	NDUFS2	0.326	0.378	0.327	0.441	0.057	0.950	-0.261	0.396	NA	NA
	NM_004551	NDUFS3	0.257	0.549	0.484	0.232	-0.212	0.674	0.694	0.012	NA	NA
	NM_002495	NDUFS4	-0.130	0.831	0.653	0.113	-0.241	0.616	0.468	0.123	NA	NA
	NM_004552	NDUFS5	-0.170	0.653	0.789	0.009	-0.272	0.525	0.219	0.504	NA	NA
	NM_004553	NDUFS6	0.387	0.447	0.417	0.342	-0.516	0.135	0.792	0.002	NA	NA
	NM_024407	NDUFS7	0.540	0.370	0.798	0.052	-0.365	0.353	0.912	0.000	NA	NA
	NM_002496	NDUFS8	1.332	0.000	0.617	0.101	-0.377	0.326	0.484	0.090	NA	NA
	NM_001166102	NDUFV1	0.453	0.206	0.415	0.290	-0.058	0.951	0.492	0.067	NA	NA
	NM_021074	NDUFV2	-0.187	0.671	0.753	0.026	0.075	0.925	-0.061	0.890	NA	NA
	NM_021075	NDUFV3	0.362	0.435	0.095	0.927	-0.187	0.739	0.936	0.000	NA	NA
	NM_004589	SCO1	0.271	0.589	-0.010	1.000	-0.070	0.943	0.642	0.022	NA	NA
	NM_001294332	SDHA	-0.128	0.780	0.195	0.702	0.350	0.357	0.196	0.543	NA	NA
	NM_003000	SDHB	0.405	0.259	0.365	0.371	-0.206	0.677	0.519	0.059	NA	NA
	NM_003001	SDHC	-0.243	0.615	-0.193	0.766	-0.212	0.675	-0.124	0.744	NA	NA
	NM_001276503	SDHD	-0.316	0.412	0.333	0.449	-0.125	0.843	0.369	0.221	NA	NA
	NM_001282198	SLC25A14	-0.128	0.829	0.960	0.018	-0.277	0.608	0.580	0.058	NA	NA
	NM_001204052	SLC25A27	-0.573	0.134	0.326	0.560	-0.055	0.968	-0.098	0.811	NA	NA
	NM_002635	SLC25A3	-0.534	0.158	0.332	0.344	-0.253	0.562	-0.122	0.716	NA	NA
	NM_001151	SLC25A4	0.198	0.607	0.506	0.149	0.017	1.000	0.354	0.207	NA	NA
	NM_001152	SLC25A5	-0.069	0.880	0.313	0.393	-0.120	0.845	-0.130	0.697	NA	NA
	NM_003172	SURF1	-0.108	0.866	0.382	0.406	0.099	0.890	0.465	0.101	NA	NA

	NM_003355	UCP2	1.016	0.025	0.950	0.012	-0.534	0.179	-0.823	0.017	NA	NA
	NM_013387	UQCR10	0.677	0.043	0.601	0.087	-0.047	0.967	0.223	0.489	NA	NA
	NM_006830	UQCR11	1.298	0.000	0.454	0.219	-0.292	0.474	0.263	0.389	NA	NA
	NM_006294	UQCRB	-0.241	0.469	0.317	0.385	-0.510	0.116	0.498	0.064	NA	NA
	NM_003365	UQCRC1	0.652	0.041	0.440	0.223	0.092	0.896	0.529	0.046	NA	NA
	NM_003366	UQCRC2	-0.122	0.774	0.123	0.821	-0.102	0.885	-0.115	0.745	NA	NA
	NM_006003	UQCRFS1	-0.099	0.851	0.532	0.153	0.049	0.966	0.772	0.003	NA	NA
	NM_001297566	UQCRH	0.686	0.050	0.604	0.096	-0.282	0.536	-0.157	0.663	NA	NA
	NM_014402	UQCRQ	0.337	0.294	0.801	0.008	-0.503	0.115	0.662	0.012	NA	NA
G25+Olea+Palm	NM_001001937	ATP5A1	NA	NA	NA	NA	-0.206	0.669	NA	NA	-0.364	0.471
	NM_001686	ATP5B	NA	NA	NA	NA	0.041	0.966	NA	NA	-0.025	1.000
	NM_005174	ATP5C1	NA	NA	NA	NA	-0.024	0.988	NA	NA	-0.219	0.823
	NM_001687	ATP5D	NA	NA	NA	NA	0.059	0.949	NA	NA	-0.222	0.842
	NM_001688	ATP5F1	NA	NA	NA	NA	-0.114	0.858	NA	NA	-0.221	0.826
	NM_005175	ATP5G1	NA	NA	NA	NA	0.138	0.822	NA	NA	0.068	1.000
	NM_001002031	ATP5G2	NA	NA	NA	NA	-0.393	0.277	NA	NA	-0.294	0.660
	NM_001689	ATP5G3	NA	NA	NA	NA	-0.062	0.943	NA	NA	-0.231	0.802
	NM_006356	ATP5H	NA	NA	NA	NA	-0.176	0.747	NA	NA	-0.216	0.840
	NM_007100	ATP5I	NA	NA	NA	NA	-0.149	0.805	NA	NA	-0.022	1.000
	NM_004889	ATP5J2	NA	NA	NA	NA	-0.204	0.687	NA	NA	-0.023	1.000
	NM_006476	ATP5L	NA	NA	NA	NA	-0.361	0.332	NA	NA	-0.142	0.941
	NM_001697	ATP5O	NA	NA	NA	NA	-0.003	1.000	NA	NA	-0.021	1.000
	NM_001003805	ATP5S	NA	NA	NA	NA	-0.248	0.675	NA	NA	-0.092	1.000
	NM_178190	ATPIF1	NA	NA	NA	NA	-0.141	0.811	NA	NA	-0.142	0.943
	NM_001162862	COX11	NA	NA	NA	NA	0.124	0.867	NA	NA	-0.038	1.000
	NM_078470	COX15	NA	NA	NA	NA	0.060	0.946	NA	NA	-0.177	0.908
	NM_005694	COX17	NA	NA	NA	NA	-0.110	0.867	NA	NA	-0.195	0.870
	NM_001861	COX4I1	NA	NA	NA	NA	0.049	0.958	NA	NA	-0.137	0.945

	NM_004255	COX5A	NA	NA	NA	NA	-0.017	1.000	NA	NA	-0.337	0.596
	NM_001862	COX5B	NA	NA	NA	NA	-0.298	0.473	NA	NA	-0.234	0.790
	NM_004373	COX6A1	NA	NA	NA	NA	-0.177	0.744	NA	NA	-0.257	0.754
	NM_001863	COX6B1	NA	NA	NA	NA	-0.085	0.908	NA	NA	-0.300	0.667
	NM_004374	COX6C	NA	NA	NA	NA	-0.388	0.287	NA	NA	-0.540	0.125
	NM_001864	COX7A1	NA	NA	NA	NA	-0.406	0.337	NA	NA	0.014	1.000
	NM_001865	COX7A2	NA	NA	NA	NA	-0.194	0.701	NA	NA	-0.120	0.968
	NM_004718	COX7A2L	NA	NA	NA	NA	-0.200	0.686	NA	NA	0.035	1.000
	NM_001866	COX7B	NA	NA	NA	NA	-0.255	0.576	NA	NA	-0.566	0.102
	NM_001867	COX7C	NA	NA	NA	NA	-0.316	0.438	NA	NA	-0.443	0.301
	NM_004074	COX8A	NA	NA	NA	NA	-0.404	0.256	NA	NA	-0.127	0.963
	NM_004541	NDUFA1	NA	NA	NA	NA	-0.198	0.700	NA	NA	-0.271	0.719
	NM_004544	NDUFA10	NA	NA	NA	NA	-0.005	1.000	NA	NA	-0.023	1.000
	NM_001258338	NDUFA12	NA	NA	NA	NA	-0.024	0.989	NA	NA	0.079	1.000
	NM_002488	NDUFA2	NA	NA	NA	NA	-0.180	0.746	NA	NA	0.019	1.000
	NM_004542	NDUFA3	NA	NA	NA	NA	-0.209	0.687	NA	NA	-0.212	0.849
	NM_002489	NDUFA4	NA	NA	NA	NA	0.035	0.976	NA	NA	-0.485	0.240
	NM_001291304	NDUFA5	NA	NA	NA	NA	-0.088	0.899	NA	NA	0.042	1.000
	NM_002490	NDUFA6	NA	NA	NA	NA	0.103	0.875	NA	NA	0.106	0.982
	NM_005001	NDUFA7	NA	NA	NA	NA	-0.507	0.132	NA	NA	-0.320	0.606
	NM_014222	NDUFA8	NA	NA	NA	NA	-0.021	0.994	NA	NA	-0.044	1.000
	NM_005002	NDUFA9	NA	NA	NA	NA	-0.009	1.000	NA	NA	-0.038	1.000
	NM_005003	NDUFAB1	NA	NA	NA	NA	-0.097	0.887	NA	NA	-0.042	1.000
	NM_004545	NDUFB1	NA	NA	NA	NA	-0.397	0.309	NA	NA	-0.537	0.149
	NM_004548	NDUFB10	NA	NA	NA	NA	-0.143	0.808	NA	NA	0.048	1.000
	NM_004546	NDUFB2	NA	NA	NA	NA	-0.345	0.368	NA	NA	0.036	1.000
	NM_002491	NDUFB3	NA	NA	NA	NA	-0.273	0.555	NA	NA	-0.513	0.183
	NM_004547	NDUFB4	NA	NA	NA	NA	-0.119	0.849	NA	NA	-0.378	0.457

NM_001199958	NDUFB5	NA	NA	NA	NA	-0.150	0.796	NA	NA	0.009	1.000
NM_002493	NDUFB6	NA	NA	NA	NA	-0.062	0.942	NA	NA	-0.279	0.700
NM_004146	NDUFB7	NA	NA	NA	NA	-0.258	0.567	NA	NA	0.062	1.000
NM_005004	NDUFB8	NA	NA	NA	NA	-0.359	0.334	NA	NA	0.011	1.000
NM_005005	NDUFB9	NA	NA	NA	NA	-0.245	0.595	NA	NA	-0.069	1.000
NM_001184990	NDUFC1	NA	NA	NA	NA	-0.138	0.818	NA	NA	-0.301	0.649
NM_001204055	NDUFC2	NA	NA	NA	NA	-0.143	0.809	NA	NA	-0.066	1.000
NM_001199981	NDUFS1	NA	NA	NA	NA	0.096	0.890	NA	NA	0.003	1.000
NM_004550	NDUFS2	NA	NA	NA	NA	0.180	0.732	NA	NA	-0.101	0.984
NM_004551	NDUFS3	NA	NA	NA	NA	-0.084	0.913	NA	NA	-0.084	1.000
NM_002495	NDUFS4	NA	NA	NA	NA	-0.241	0.625	NA	NA	-0.120	0.974
NM_004552	NDUFS5	NA	NA	NA	NA	0.015	1.000	NA	NA	-0.273	0.715
NM_004553	NDUFS6	NA	NA	NA	NA	-0.089	0.907	NA	NA	-0.018	1.000
NM_024407	NDUFS7	NA	NA	NA	NA	-0.232	0.642	NA	NA	0.079	1.000
NM_002496	NDUFS8	NA	NA	NA	NA	-0.246	0.607	NA	NA	-0.237	0.810
NM_001166102	NDUFV1	NA	NA	NA	NA	-0.121	0.847	NA	NA	-0.138	0.951
NM_021074	NDUFV2	NA	NA	NA	NA	0.134	0.821	NA	NA	0.001	1.000
NM_021075	NDUFV3	NA	NA	NA	NA	-0.092	0.903	NA	NA	-0.010	1.000
NM_004589	SCO1	NA	NA	NA	NA	0.276	0.573	NA	NA	-0.003	1.000
NM_001294332	SDHA	NA	NA	NA	NA	0.226	0.638	NA	NA	-0.043	1.000
NM_003000	SDHB	NA	NA	NA	NA	-0.011	1.000	NA	NA	-0.131	0.963
NM_003001	SDHC	NA	NA	NA	NA	-0.013	1.000	NA	NA	-0.153	0.932
NM_001276503	SDHD	NA	NA	NA	NA	-0.060	0.946	NA	NA	0.082	1.000
NM_001282198	SLC25A14	NA	NA	NA	NA	-0.315	0.545	NA	NA	0.097	0.996
NM_001204052	SLC25A27	NA	NA	NA	NA	-0.068	0.950	NA	NA	0.056	1.000
NM_002635	SLC25A3	NA	NA	NA	NA	-0.034	0.976	NA	NA	-0.327	0.573
NM_001151	SLC25A4	NA	NA	NA	NA	-0.201	0.687	NA	NA	0.082	1.000
NM_001152	SLC25A5	NA	NA	NA	NA	-0.080	0.912	NA	NA	0.118	0.970

	NM_003172	SURF1	NA	NA	NA	NA	-0.022	0.992	NA	NA	0.203	0.868
	NM_003355	UCP2	NA	NA	NA	NA	-0.047	0.968	NA	NA	0.333	0.596
	NM_013387	UQCR10	NA	NA	NA	NA	-0.002	1.000	NA	NA	0.038	1.000
	NM_006830	UQCR11	NA	NA	NA	NA	-0.117	0.849	NA	NA	-0.035	1.000
	NM_006294	UQCRB	NA	NA	NA	NA	-0.290	0.494	NA	NA	-0.241	0.779
	NM_003365	UQCRC1	NA	NA	NA	NA	-0.045	0.963	NA	NA	0.012	1.000
	NM_003366	UQCRC2	NA	NA	NA	NA	-0.140	0.816	NA	NA	-0.024	1.000
	NM_006003	UQCRFS1	NA	NA	NA	NA	0.159	0.784	NA	NA	-0.047	1.000
	NM_001297566	UQCRH	NA	NA	NA	NA	-0.135	0.838	NA	NA	-0.303	0.681
	NM_014402	UQCRQ	NA	NA	NA	NA	-0.307	0.446	NA	NA	-0.082	0.998
G25+Olea	NM_001001937	ATP5A1	NA	NA	NA	NA	-0.303	0.420	-0.323	0.227	-0.409	0.273
	NM_001686	ATP5B	NA	NA	NA	NA	-0.003	1.000	0.967	0.000	-0.061	0.966
	NM_005174	ATP5C1	NA	NA	NA	NA	-0.007	1.000	-0.338	0.225	-0.062	0.966
	NM_001687	ATP5D	NA	NA	NA	NA	-0.046	0.962	0.592	0.024	-0.279	0.642
	NM_001688	ATP5F1	NA	NA	NA	NA	-0.246	0.566	0.420	0.117	-0.031	0.998
	NM_005175	ATP5G1	NA	NA	NA	NA	-0.100	0.878	1.073	0.000	0.024	1.000
	NM_001002031	ATP5G2	NA	NA	NA	NA	-0.487	0.124	-0.709	0.005	-0.257	0.649
	NM_001689	ATP5G3	NA	NA	NA	NA	-0.200	0.668	0.438	0.094	-0.163	0.845
	NM_006356	ATP5H	NA	NA	NA	NA	-0.326	0.394	0.221	0.482	-0.247	0.684
	NM_007100	ATP5I	NA	NA	NA	NA	-0.233	0.601	0.787	0.009	-0.082	0.952
	NM_004889	ATP5J2	NA	NA	NA	NA	-0.193	0.685	1.213	0.000	-0.107	0.926
	NM_006476	ATP5L	NA	NA	NA	NA	-0.439	0.177	-0.435	0.097	-0.212	0.748
	NM_001697	ATP5O	NA	NA	NA	NA	-0.086	0.894	0.030	0.946	0.036	0.994
	NM_001003805	ATP5S	NA	NA	NA	NA	-0.166	0.791	0.023	0.984	-0.274	0.687
	NM_178190	ATPIF1	NA	NA	NA	NA	-0.070	0.923	0.894	0.000	-0.079	0.953
	NM_001162862	COX11	NA	NA	NA	NA	-0.155	0.793	0.737	0.004	-0.205	0.802
	NM_078470	COX15	NA	NA	NA	NA	0.145	0.793	0.255	0.372	-0.271	0.649
	NM_005694	COX17	NA	NA	NA	NA	-0.341	0.358	-0.947	0.000	-0.187	0.798

	NM_001861	COX4I1	NA	NA	NA	NA	-0.039	0.967	-0.114	0.714	-0.124	0.896
	NM_004255	COX5A	NA	NA	NA	NA	-0.167	0.750	-0.097	0.784	-0.292	0.612
	NM_001862	COX5B	NA	NA	NA	NA	-0.356	0.323	0.452	0.093	-0.066	0.962
	NM_004373	COX6A1	NA	NA	NA	NA	-0.370	0.303	0.563	0.029	-0.298	0.566
	NM_001863	COX6B1	NA	NA	NA	NA	-0.208	0.654	0.447	0.096	-0.159	0.856
	NM_004374	COX6C	NA	NA	NA	NA	-0.548	0.069	-0.512	0.063	-0.267	0.624
	NM_001864	COX7A1	NA	NA	NA	NA	-0.271	0.567	-0.332	0.403	-0.087	0.956
	NM_001865	COX7A2	NA	NA	NA	NA	-0.280	0.477	-0.612	0.017	-0.111	0.918
	NM_004718	COX7A2L	NA	NA	NA	NA	-0.089	0.885	0.218	0.444	0.095	0.935
	NM_001866	COX7B	NA	NA	NA	NA	-0.444	0.181	-0.680	0.014	-0.375	0.371
	NM_001867	COX7C	NA	NA	NA	NA	-0.393	0.257	-0.256	0.363	-0.333	0.468
	NM_004074	COX8A	NA	NA	NA	NA	-0.486	0.124	0.045	0.903	-0.197	0.787
	NM_004541	NDUFA1	NA	NA	NA	NA	-0.359	0.323	0.317	0.264	-0.295	0.565
	NM_004544	NDUFA10	NA	NA	NA	NA	-0.108	0.858	0.721	0.004	-0.091	0.944
	NM_001258338	NDUFA12	NA	NA	NA	NA	-0.057	0.945	0.194	0.532	0.044	0.985
	NM_002488	NDUFA2	NA	NA	NA	NA	-0.096	0.883	0.175	0.578	0.113	0.921
	NM_004542	NDUFA3	NA	NA	NA	NA	-0.209	0.662	0.611	0.035	-0.412	0.306
	NM_002489	NDUFA4	NA	NA	NA	NA	-0.188	0.698	0.770	0.003	-0.170	0.841
	NM_001291304	NDUFA5	NA	NA	NA	NA	-0.123	0.822	0.338	0.217	0.077	0.953
	NM_002490	NDUFA6	NA	NA	NA	NA	0.057	0.942	1.371	0.000	0.135	0.884
	NM_005001	NDUFA7	NA	NA	NA	NA	-0.663	0.024	0.117	0.763	-0.166	0.845
	NM_014222	NDUFA8	NA	NA	NA	NA	-0.169	0.745	0.535	0.044	-0.124	0.902
	NM_005002	NDUFA9	NA	NA	NA	NA	-0.200	0.668	0.531	0.044	0.053	0.978
	NM_005003	NDUFAB1	NA	NA	NA	NA	-0.170	0.735	0.362	0.196	0.013	1.000
	NM_004545	NDUFB1	NA	NA	NA	NA	-0.442	0.206	-0.095	0.848	-0.427	0.274
	NM_004548	NDUFB10	NA	NA	NA	NA	-0.099	0.872	0.687	0.007	-0.202	0.785
	NM_004546	NDUFB2	NA	NA	NA	NA	-0.302	0.428	-0.002	1.000	-0.065	0.962
	NM_002491	NDUFB3	NA	NA	NA	NA	-0.469	0.163	0.529	0.075	-0.383	0.365

	NM_004547	NDUFB4	NA	NA	NA	NA	-0.330	0.380	0.543	0.034	-0.270	0.625
	NM_001199958	NDUFB5	NA	NA	NA	NA	-0.162	0.753	0.048	0.900	0.097	0.935
	NM_002493	NDUFB6	NA	NA	NA	NA	-0.214	0.639	0.370	0.195	-0.203	0.778
	NM_004146	NDUFB7	NA	NA	NA	NA	-0.245	0.568	0.978	0.000	-0.116	0.913
	NM_005004	NDUFB8	NA	NA	NA	NA	-0.410	0.218	-0.311	0.257	-0.119	0.903
	NM_005005	NDUFB9	NA	NA	NA	NA	-0.262	0.526	1.288	0.000	0.095	0.937
	NM_001184990	NDUFC1	NA	NA	NA	NA	-0.199	0.673	0.624	0.026	-0.228	0.722
	NM_001204055	NDUFC2	NA	NA	NA	NA	-0.089	0.892	-0.372	0.207	-0.073	0.959
	NM_001199981	NDUFS1	NA	NA	NA	NA	-0.016	1.000	0.073	0.831	0.001	1.000
	NM_004550	NDUFS2	NA	NA	NA	NA	0.133	0.806	0.107	0.743	-0.116	0.911
	NM_004551	NDUFS3	NA	NA	NA	NA	-0.312	0.434	0.827	0.001	-0.269	0.641
	NM_002495	NDUFS4	NA	NA	NA	NA	-0.236	0.606	0.216	0.505	0.062	0.968
	NM_004552	NDUFS5	NA	NA	NA	NA	-0.142	0.791	0.342	0.234	-0.074	0.959
	NM_004553	NDUFS6	NA	NA	NA	NA	-0.332	0.401	1.107	0.000	-0.194	0.801
	NM_024407	NDUFS7	NA	NA	NA	NA	-0.338	0.381	1.321	0.000	-0.267	0.662
	NM_002496	NDUFS8	NA	NA	NA	NA	-0.229	0.611	0.799	0.002	-0.351	0.460
	NM_001166102	NDUFV1	NA	NA	NA	NA	0.042	0.966	0.805	0.001	-0.193	0.801
	NM_021074	NDUFV2	NA	NA	NA	NA	0.105	0.859	-0.007	1.000	0.165	0.840
	NM_021075	NDUFV3	NA	NA	NA	NA	-0.076	0.922	1.139	0.000	-0.117	0.921
	NM_004589	SCO1	NA	NA	NA	NA	0.093	0.901	0.756	0.004	0.204	0.797
	NM_001294332	SDHA	NA	NA	NA	NA	0.528	0.084	0.265	0.352	-0.149	0.872
	NM_003000	SDHB	NA	NA	NA	NA	-0.105	0.861	0.428	0.113	-0.067	0.962
	NM_003001	SDHC	NA	NA	NA	NA	-0.114	0.847	0.247	0.421	-0.036	0.994
	NM_001276503	SDHD	NA	NA	NA	NA	-0.033	0.980	0.340	0.238	0.181	0.815
	NM_001282198	SLC25A14	NA	NA	NA	NA	-0.641	0.072	1.188	0.000	0.054	0.983
	NM_001204052	SLC25A27	NA	NA	NA	NA	-0.122	0.883	-0.075	0.854	0.188	0.894
	NM_002635	SLC25A3	NA	NA	NA	NA	-0.076	0.912	-0.026	0.949	-0.259	0.644
	NM_001151	SLC25A4	NA	NA	NA	NA	-0.203	0.656	0.282	0.305	-0.106	0.925

	NM_001152	SLC25A5	NA	NA	NA	NA	0.003	1.000	-0.304	0.262	-0.008	1.000
	NM_003172	SURF1	NA	NA	NA	NA	0.025	0.990	0.841	0.001	0.106	0.933
	NM_003355	UCP2	NA	NA	NA	NA	0.215	0.696	0.306	0.370	0.005	1.000
	NM_013387	UQCR10	NA	NA	NA	NA	0.025	0.989	0.503	0.059	0.052	0.977
	NM_006830	UQCR11	NA	NA	NA	NA	-0.136	0.798	0.474	0.071	0.045	0.983
	NM_006294	UQCRB	NA	NA	NA	NA	-0.452	0.169	0.513	0.045	-0.101	0.933
	NM_003365	UQCRC1	NA	NA	NA	NA	0.035	0.973	0.497	0.052	-0.135	0.884
	NM_003366	UQCRC2	NA	NA	NA	NA	-0.098	0.877	-0.352	0.197	0.053	0.978
	NM_006003	UQCRFS1	NA	NA	NA	NA	0.096	0.881	0.827	0.001	0.102	0.934
	NM_001297566	UQCRH	NA	NA	NA	NA	-0.237	0.612	-0.201	0.526	0.032	0.998
	NM_014402	UQCRQ	NA	NA	NA	NA	-0.380	0.270	0.966	0.000	-0.040	0.988
G25+Palm	NM_001001937	ATP5A1	NA	NA	NA	NA	-0.310	0.355	NA	NA	-0.285	0.560
	NM_001686	ATP5B	NA	NA	NA	NA	-0.169	0.691	NA	NA	0.011	1.000
	NM_005174	ATP5C1	NA	NA	NA	NA	0.028	0.975	NA	NA	-0.116	0.902
	NM_001687	ATP5D	NA	NA	NA	NA	-0.139	0.778	NA	NA	0.031	1.000
	NM_001688	ATP5F1	NA	NA	NA	NA	-0.093	0.866	NA	NA	-0.026	1.000
	NM_005175	ATP5G1	NA	NA	NA	NA	-0.212	0.618	NA	NA	0.280	0.598
	NM_001002031	ATP5G2	NA	NA	NA	NA	-0.366	0.254	NA	NA	-0.263	0.627
	NM_001689	ATP5G3	NA	NA	NA	NA	-0.072	0.903	NA	NA	0.028	1.000
	NM_006356	ATP5H	NA	NA	NA	NA	-0.154	0.738	NA	NA	-0.295	0.560
	NM_007100	ATP5I	NA	NA	NA	NA	-0.003	1.000	NA	NA	0.235	0.701
	NM_004889	ATP5J2	NA	NA	NA	NA	-0.208	0.610	NA	NA	0.021	1.000
	NM_006476	ATP5L	NA	NA	NA	NA	-0.365	0.252	NA	NA	-0.031	1.000
	NM_001697	ATP5O	NA	NA	NA	NA	0.090	0.871	NA	NA	0.101	0.923
	NM_001003805	ATP5S	NA	NA	NA	NA	-0.150	0.797	NA	NA	-0.267	0.690
	NM_178190	ATPIF1	NA	NA	NA	NA	-0.322	0.344	NA	NA	-0.116	0.903
	NM_001162862	COX11	NA	NA	NA	NA	-0.310	0.433	NA	NA	0.123	0.906
	NM_078470	COX15	NA	NA	NA	NA	0.170	0.710	NA	NA	-0.172	0.832

	NM_005694	COX17	NA	NA	NA	NA	-0.177	0.682	NA	NA	-0.081	0.952
	NM_001861	COX4I1	NA	NA	NA	NA	0.085	0.880	NA	NA	0.069	0.967
	NM_004255	COX5A	NA	NA	NA	NA	-0.098	0.861	NA	NA	-0.085	0.953
	NM_001862	COX5B	NA	NA	NA	NA	-0.191	0.647	NA	NA	-0.142	0.865
	NM_004373	COX6A1	NA	NA	NA	NA	-0.216	0.595	NA	NA	-0.236	0.701
	NM_001863	COX6B1	NA	NA	NA	NA	-0.061	0.925	NA	NA	-0.038	0.994
	NM_004374	COX6C	NA	NA	NA	NA	-0.293	0.401	NA	NA	-0.169	0.820
	NM_001864	COX7A1	NA	NA	NA	NA	-0.022	0.988	NA	NA	0.292	0.622
	NM_001865	COX7A2	NA	NA	NA	NA	-0.116	0.818	NA	NA	0.092	0.935
	NM_004718	COX7A2L	NA	NA	NA	NA	0.025	0.977	NA	NA	0.104	0.917
	NM_001866	COX7B	NA	NA	NA	NA	-0.157	0.730	NA	NA	-0.219	0.730
	NM_001867	COX7C	NA	NA	NA	NA	-0.051	0.940	NA	NA	-0.149	0.855
	NM_004074	COX8A	NA	NA	NA	NA	-0.539	0.057	NA	NA	-0.049	0.982
	NM_004541	NDUFA1	NA	NA	NA	NA	-0.310	0.373	NA	NA	-0.158	0.841
	NM_004544	NDUFA10	NA	NA	NA	NA	-0.164	0.718	NA	NA	-0.184	0.804
	NM_001258338	NDUFA12	NA	NA	NA	NA	0.049	0.944	NA	NA	0.136	0.875
	NM_002488	NDUFA2	NA	NA	NA	NA	-0.138	0.781	NA	NA	0.144	0.870
	NM_004542	NDUFA3	NA	NA	NA	NA	-0.278	0.458	NA	NA	-0.025	1.000
	NM_002489	NDUFA4	NA	NA	NA	NA	0.052	0.940	NA	NA	-0.072	0.967
	NM_001291304	NDUFA5	NA	NA	NA	NA	-0.069	0.910	NA	NA	0.141	0.864
	NM_002490	NDUFA6	NA	NA	NA	NA	0.228	0.556	NA	NA	0.190	0.782
	NM_005001	NDUFA7	NA	NA	NA	NA	-0.576	0.046	NA	NA	-0.055	0.981
	NM_014222	NDUFA8	NA	NA	NA	NA	0.002	1.000	NA	NA	-0.096	0.936
	NM_005002	NDUFA9	NA	NA	NA	NA	-0.059	0.926	NA	NA	0.086	0.946
	NM_005003	NDUFAB1	NA	NA	NA	NA	-0.208	0.612	NA	NA	-0.078	0.957
	NM_004545	NDUFB1	NA	NA	NA	NA	-0.515	0.093	NA	NA	-0.299	0.560
	NM_004548	NDUFB10	NA	NA	NA	NA	-0.205	0.619	NA	NA	0.086	0.949
	NM_004546	NDUFB2	NA	NA	NA	NA	-0.391	0.212	NA	NA	0.093	0.934

	NM_002491	NDUFB3	NA	NA	NA	NA	-0.369	0.273	NA	NA	-0.211	0.752
	NM_004547	NDUFB4	NA	NA	NA	NA	-0.250	0.505	NA	NA	-0.159	0.842
	NM_001199958	NDUFB5	NA	NA	NA	NA	-0.255	0.497	NA	NA	0.126	0.889
	NM_002493	NDUFB6	NA	NA	NA	NA	-0.215	0.596	NA	NA	-0.098	0.927
	NM_004146	NDUFB7	NA	NA	NA	NA	-0.358	0.275	NA	NA	0.116	0.905
	NM_005004	NDUFB8	NA	NA	NA	NA	-0.289	0.405	NA	NA	0.098	0.925
	NM_005005	NDUFB9	NA	NA	NA	NA	-0.221	0.575	NA	NA	0.029	1.000
	NM_001184990	NDUFC1	NA	NA	NA	NA	-0.061	0.924	NA	NA	-0.204	0.761
	NM_001204055	NDUFC2	NA	NA	NA	NA	-0.153	0.741	NA	NA	0.136	0.875
	NM_001199981	NDUFS1	NA	NA	NA	NA	-0.083	0.888	NA	NA	0.047	0.985
	NM_004550	NDUFS2	NA	NA	NA	NA	0.030	0.972	NA	NA	-0.084	0.950
	NM_004551	NDUFS3	NA	NA	NA	NA	-0.266	0.485	NA	NA	-0.041	0.990
	NM_002495	NDUFS4	NA	NA	NA	NA	0.113	0.838	NA	NA	0.053	0.983
	NM_004552	NDUFS5	NA	NA	NA	NA	-0.029	0.974	NA	NA	-0.034	0.997
	NM_004553	NDUFS6	NA	NA	NA	NA	-0.131	0.798	NA	NA	0.176	0.819
	NM_024407	NDUFS7	NA	NA	NA	NA	-0.499	0.102	NA	NA	0.068	0.972
	NM_002496	NDUFS8	NA	NA	NA	NA	-0.452	0.149	NA	NA	-0.064	0.974
	NM_001166102	NDUFV1	NA	NA	NA	NA	-0.147	0.754	NA	NA	-0.018	1.000
	NM_021074	NDUFV2	NA	NA	NA	NA	0.335	0.316	NA	NA	0.361	0.391
	NM_021075	NDUFV3	NA	NA	NA	NA	-0.021	0.985	NA	NA	0.119	0.908
	NM_004589	SCO1	NA	NA	NA	NA	-0.120	0.835	NA	NA	0.017	1.000
	NM_001294332	SDHA	NA	NA	NA	NA	0.241	0.525	NA	NA	-0.084	0.952
	NM_003000	SDHB	NA	NA	NA	NA	-0.084	0.886	NA	NA	0.032	1.000
	NM_003001	SDHC	NA	NA	NA	NA	-0.365	0.277	NA	NA	-0.257	0.660
	NM_001276503	SDHD	NA	NA	NA	NA	-0.001	1.000	NA	NA	0.186	0.795
	NM_001282198	SLC25A14	NA	NA	NA	NA	-0.427	0.251	NA	NA	0.260	0.699
	NM_001204052	SLC25A27	NA	NA	NA	NA	-0.106	0.880	NA	NA	0.250	0.814
	NM_002635	SLC25A3	NA	NA	NA	NA	-0.019	0.985	NA	NA	-0.215	0.736

NM_001151	SLC25A4	NA	NA	NA	NA	-0.135	0.774	NA	NA	-0.023	1.000
NM_001152	SLC25A5	NA	NA	NA	NA	-0.013	0.996	NA	NA	0.210	0.742
NM_003172	SURF1	NA	NA	NA	NA	0.093	0.869	NA	NA	0.282	0.604
NM_003355	UCP2	NA	NA	NA	NA	-0.751	0.017	NA	NA	0.594	0.068
NM_013387	UQCR10	NA	NA	NA	NA	0.073	0.901	NA	NA	0.089	0.940
NM_006830	UQCR11	NA	NA	NA	NA	-0.263	0.468	NA	NA	0.044	0.986
NM_006294	UQCRB	NA	NA	NA	NA	-0.164	0.712	NA	NA	-0.073	0.963
NM_003365	UQCRC1	NA	NA	NA	NA	0.055	0.930	NA	NA	-0.042	0.988
NM_003366	UQCRC2	NA	NA	NA	NA	-0.063	0.922	NA	NA	0.033	0.999
NM_006003	UQCRFS1	NA	NA	NA	NA	0.029	0.976	NA	NA	0.131	0.887
NM_001297566	UQCRH	NA	NA	NA	NA	0.044	0.958	NA	NA	0.126	0.899
NM_014402	UQCRQ	NA	NA	NA	NA	-0.197	0.628	NA	NA	0.121	0.893

Supplementary Table S4. Quantitative data related to the transcriptomic profiles of the outer and inner mitochondrial membrane translocases TOM/TIM machinery in human islets upon metabolic stress.NA: Not applicable (condition not tested); ND: not detected.

			**Donor #1**		**Donor #2**		**Donor #3**		**Donor #4**		**Donor #5**	
	Accession (NCBI Ref. Sequence)	Gene symbol	**Log$_2$ FC**	**Adjusted *p***	**Log$_2$ FC**	**Adjusted *p***	**Log$_2$ FC**	**Adjusted *p***	**Log$_2$ FC**	**Adjusted *p***	**Log$_2$ FC**	**Adjusted *p***
Olea	NM_012456	TIMM10	0.302	0.646	0.666	0.191	NA	NA	0.500	0.215	NA	NA
	NM_012192	TIMM10B	0.228	0.598	-0.219	0.675	NA	NA	-0.044	0.956	NA	NA
	NM_012458	TIMM13	1.084	0.020	0.574	0.198	NA	NA	0.214	0.623	NA	NA
	NM_006335	TIMM17A	-0.476	0.209	-0.016	1.000	NA	NA	-0.394	0.265	NA	NA
	NM_005834	TIMM17B	1.590	0.007	0.639	0.183	NA	NA	0.287	0.480	NA	NA
	NM_014177	TIMM21	-0.565	0.208	-0.059	0.966	NA	NA	-0.461	0.234	NA	NA
	NM_013337	TIMM22	0.499	0.297	0.338	0.528	NA	NA	0.048	0.954	NA	NA
	NM_006327	TIMM23	-0.657	0.080	0.600	0.183	NA	NA	-0.081	0.915	NA	NA

NM_001290117	TIMM23B	ND	ND	ND	ND	NA	NA	-0.394	0.577	NA	NA
NM_006351	TIMM44	0.911	0.045	0.083	0.933	NA	NA	-0.073	0.911	NA	NA
NM_001001563	TIMM50	0.602	0.225	0.369	0.507	NA	NA	-0.142	0.791	NA	NA
NM_004085	TIMM8A	-0.075	1.000	-0.073	1.000	NA	NA	-0.865	0.080	NA	NA
NM_012459	TIMM8B	-0.188	0.826	0.127	0.886	NA	NA	-0.168	0.772	NA	NA
NM_001304485	TIMM9	-0.601	0.204	0.055	0.969	NA	NA	-0.359	0.458	NA	NA
NM_016589	TIMMDC1	-0.188	0.689	0.070	0.932	NA	NA	-0.047	0.952	NA	NA
NM_014765	TOMM20	-0.188	0.629	0.076	0.913	NA	NA	-0.380	0.264	NA	NA
NM_020243	TOMM22	-0.020	1.000	0.158	0.793	NA	NA	0.113	0.843	NA	NA
NM_006809	TOMM34	-0.048	0.949	0.044	0.969	NA	NA	0.002	1.000	NA	NA
NM_006114	TOMM40	1.395	0.036	0.800	0.113	NA	NA	-0.010	1.000	NA	NA
NM_001286373	TOMM40L	0.572	0.375	0.254	0.767	NA	NA	0.174	0.791	NA	NA
NM_001134485	TOMM5	-0.277	0.513	0.421	0.354	NA	NA	0.217	0.636	NA	NA
NM_001134493	TOMM6	-0.365	0.350	-0.057	0.949	NA	NA	-0.068	0.918	NA	NA
NM_019059	TOMM7	-0.146	0.797	0.153	0.807	NA	NA	0.141	0.783	NA	NA
NM_014820	TOMM70A	-0.177	0.657	-0.068	0.930	NA	NA	-0.324	0.378	NA	NA
NM_012456	TIMM10	0.353	0.618	-0.207	1.000	NA	NA	NA	NA	NA	NA
NM_012192	TIMM10B	0.378	0.350	-0.151	1.000	NA	NA	NA	NA	NA	NA
NM_012458	TIMM13	1.052	0.034	0.180	1.000	NA	NA	NA	NA	NA	NA
NM_006335	TIMM17A	0.004	1.000	-0.090	1.000	NA	NA	NA	NA	NA	NA
NM_005834	TIMM17B	1.983	0.000	0.399	1.000	NA	NA	NA	NA	NA	NA
NM_014177	TIMM21	-0.332	0.515	-0.106	1.000	NA	NA	NA	NA	NA	NA
NM_013337	TIMM22	0.572	0.245	0.060	1.000	NA	NA	NA	NA	NA	NA
NM_006327	TIMM23	-0.044	0.978	0.172	1.000	NA	NA	NA	NA	NA	NA
NM_001290117	TIMM23B	ND	ND	ND	ND	NA	NA	NA	NA	NA	NA
NM_006351	TIMM44	0.245	0.744	-0.476	1.000	NA	NA	NA	NA	NA	NA
NM_001001563	TIMM50	0.155	0.896	0.223	1.000	NA	NA	NA	NA	NA	NA
NM_004085	TIMM8A	-0.368	0.834	-0.259	1.000	NA	NA	NA	NA	NA	NA

Palm	NM_012459	TIMM8B	0.129	0.945	0.066	1.000	NA	NA	NA	NA	NA	NA
	NM_001304485	TIMM9	0.098	0.929	-0.031	1.000	NA	NA	NA	NA	NA	NA
	NM_016589	TIMMDC1	0.274	0.519	-0.152	1.000	NA	NA	NA	NA	NA	NA
	NM_014765	TOMM20	0.066	0.921	-0.012	1.000	NA	NA	NA	NA	NA	NA
	NM_020243	TOMM22	-0.006	1.000	-0.061	1.000	NA	NA	NA	NA	NA	NA
	NM_006809	TOMM34	-0.045	0.974	-0.225	1.000	NA	NA	NA	NA	NA	NA
	NM_006114	TOMM40	1.207	0.091	0.755	0.897	NA	NA	NA	NA	NA	NA
	NM_001286373	TOMM40L	0.576	0.367	-0.218	1.000	NA	NA	NA	NA	NA	NA
	NM_001134485	TOMM5	-0.252	0.599	0.276	1.000	NA	NA	NA	NA	NA	NA
	NM_001134493	TOMM6	-0.062	0.954	-0.235	1.000	NA	NA	NA	NA	NA	NA
	NM_019059	TOMM7	0.426	0.309	-0.100	1.000	NA	NA	NA	NA	NA	NA
	NM_014820	TOMM70A	-0.177	0.691	0.068	1.000	NA	NA	NA	NA	NA	NA
G25	NM_012456	TIMM10	0.062	0.989	0.734	0.128	-0.276	0.608	1.104	0.000	NA	NA
	NM_012192	TIMM10B	0.192	0.644	0.129	0.819	-0.233	0.619	0.783	0.002	NA	NA
	NM_012458	TIMM13	1.202	0.007	0.712	0.069	-0.356	0.359	0.317	0.282	NA	NA
	NM_006335	TIMM17A	-0.147	0.743	0.691	0.052	-0.133	0.827	0.424	0.127	NA	NA
	NM_005834	TIMM17B	2.315	0.000	0.594	0.214	-0.185	0.730	-0.210	0.529	NA	NA
	NM_014177	TIMM21	-0.931	0.020	0.414	0.411	-0.291	0.520	0.965	0.000	NA	NA
	NM_013337	TIMM22	0.605	0.147	0.623	0.142	0.049	0.969	0.793	0.002	NA	NA
	NM_006327	TIMM23	-0.269	0.516	0.763	0.046	-0.077	0.931	1.135	0.000	NA	NA
	NM_001290117	TIMM23B	ND	ND	ND	ND	-1.297	0.065	0.202	0.744	NA	NA
	NM_006351	TIMM44	0.537	0.283	0.261	0.625	-0.099	0.910	0.403	0.155	NA	NA
	NM_001001563	TIMM50	0.205	0.759	0.231	0.710	-0.157	0.807	0.972	0.000	NA	NA
	NM_004085	TIMM8A	0.262	0.797	0.610	0.565	-0.158	0.874	1.106	0.001	NA	NA
	NM_012459	TIMM8B	0.812	0.079	0.029	1.000	-0.288	0.522	0.370	0.246	NA	NA
	NM_001304485	TIMM9	-0.317	0.494	-0.288	0.629	-0.702	0.067	0.290	0.431	NA	NA
	NM_016589	TIMMDC1	0.053	0.944	0.338	0.395	0.067	0.940	0.242	0.433	NA	NA
	NM_014765	TOMM20	-0.308	0.338	-0.450	0.187	-0.166	0.772	-0.819	0.001	NA	NA

	NM_020243	TOMM22	0.140	0.764	0.259	0.557	-0.322	0.448	0.124	0.736	NA	NA
	NM_006809	TOMM34	0.297	0.409	-0.200	0.650	-0.338	0.390	-0.388	0.173	NA	NA
	NM_006114	TOMM40	2.089	0.000	0.660	0.212	0.105	0.884	0.558	0.050	NA	NA
	NM_001286373	TOMM40L	0.542	0.410	-0.105	0.965	0.121	0.920	-0.295	0.457	NA	NA
	NM_001134485	TOMM5	0.255	0.519	0.507	0.187	-0.118	0.852	-0.003	1.000	NA	NA
	NM_001134493	TOMM6	-0.118	0.791	0.436	0.241	-0.297	0.486	-0.237	0.449	NA	NA
	NM_019059	TOMM7	0.129	0.817	0.262	0.578	-0.347	0.386	-0.799	0.003	NA	NA
	NM_014820	TOMM70A	-0.416	0.175	0.047	0.962	0.709	0.018	1.253	0.000	NA	NA
G25 + Olea + Palm	NM_012456	TIMM10	NA	NA	NA	NA	NA	NA	0.049	0.968	-0.213	0.886
	NM_012192	TIMM10B	NA	NA	NA	NA	NA	NA	0.021	0.993	0.022	1.000
	NM_012458	TIMM13	NA	NA	NA	NA	NA	NA	-0.067	0.937	-0.165	0.919
	NM_006335	TIMM17A	NA	NA	NA	NA	NA	NA	0.106	0.875	-0.078	1.000
	NM_005834	TIMM17B	NA	NA	NA	NA	NA	NA	-0.022	0.991	0.021	1.000
	NM_014177	TIMM21	NA	NA	NA	NA	NA	NA	-0.126	0.851	-0.116	0.980
	NM_013337	TIMM22	NA	NA	NA	NA	NA	NA	0.216	0.673	0.053	1.000
	NM_006327	TIMM23	NA	NA	NA	NA	NA	NA	0.332	0.441	-0.011	1.000
	NM_001290117	TIMM23B	NA	NA	NA	NA	NA	NA	-0.068	0.990	0.830	0.315
	NM_006351	TIMM44	NA	NA	NA	NA	NA	NA	0.072	0.943	-0.157	0.950
	NM_001001563	TIMM50	NA	NA	NA	NA	NA	NA	0.154	0.808	-0.090	1.000
	NM_004085	TIMM8A	NA	NA	NA	NA	NA	NA	0.113	0.930	-0.072	1.000
	NM_012459	TIMM8B	NA	NA	NA	NA	NA	NA	-0.217	0.684	-0.079	1.000
	NM_001304485	TIMM9	NA	NA	NA	NA	NA	NA	-0.634	0.119	-0.420	0.555
	NM_016589	TIMMDC1	NA	NA	NA	NA	NA	NA	0.109	0.869	0.058	1.000
	NM_014765	TOMM20	NA	NA	NA	NA	NA	NA	-0.203	0.703	-0.378	0.497
	NM_020243	TOMM22	NA	NA	NA	NA	NA	NA	-0.037	0.978	-0.183	0.899
	NM_006809	TOMM34	NA	NA	NA	NA	NA	NA	-0.472	0.166	-0.462	0.264
	NM_006114	TOMM40	NA	NA	NA	NA	NA	NA	0.336	0.416	0.483	0.283
	NM_001286373	TOMM40L	NA	NA	NA	NA	NA	NA	0.167	0.859	0.058	1.000

	NM_001134485	TOMM5	NA	NA	NA	NA	NA	NA	0.118	0.851	-0.011	1.000
	NM_001134493	TOMM6	NA	NA	NA	NA	NA	NA	-0.070	0.936	-0.280	0.712
	NM_019059	TOMM7	NA	NA	NA	NA	NA	NA	-0.347	0.397	-0.200	0.870
	NM_014820	TOMM70A	NA	NA	NA	NA	NA	NA	0.675	0.029	1.027	0.000
G25 + Olea	NM_012456	TIMM10	NA	NA	NA	NA	-0.199	0.732	1.214	0.000	-0.092	0.956
	NM_012192	TIMM10B	NA	NA	NA	NA	-0.005	1.000	1.009	0.000	-0.064	0.965
	NM_012458	TIMM13	NA	NA	NA	NA	-0.194	0.687	0.812	0.001	-0.172	0.838
	NM_006335	TIMM17A	NA	NA	NA	NA	-0.002	1.000	0.448	0.089	0.000	1.000
	NM_005834	TIMM17B	NA	NA	NA	NA	-0.111	0.856	0.357	0.198	-0.072	0.960
	NM_014177	TIMM21	NA	NA	NA	NA	-0.163	0.767	0.839	0.001	-0.018	1.000
	NM_013337	TIMM22	NA	NA	NA	NA	-0.019	0.998	0.849	0.001	-0.183	0.823
	NM_006327	TIMM23	NA	NA	NA	NA	0.165	0.760	0.942	0.000	-0.062	0.969
	NM_001290117	TIMM23B	NA	NA	NA	NA	0.329	0.727	0.084	0.944	0.519	0.639
	NM_006351	TIMM44	NA	NA	NA	NA	-0.005	1.000	0.318	0.255	-0.369	0.508
	NM_001001563	TIMM50	NA	NA	NA	NA	-0.110	0.873	1.130	0.000	-0.110	0.935
	NM_004085	TIMM8A	NA	NA	NA	NA	-0.308	0.655	-0.157	0.741	-0.499	0.490
	NM_012459	TIMM8B	NA	NA	NA	NA	-0.129	0.830	0.321	0.299	0.005	1.000
	NM_001304485	TIMM9	NA	NA	NA	NA	-0.635	0.093	-0.104	0.808	-0.369	0.570
	NM_016589	TIMMDC1	NA	NA	NA	NA	0.039	0.969	0.242	0.403	0.064	0.964
	NM_014765	TOMM20	NA	NA	NA	NA	-0.295	0.477	-1.257	0.000	-0.263	0.674
	NM_020243	TOMM22	NA	NA	NA	NA	-0.171	0.748	0.551	0.037	-0.067	0.965
	NM_006809	TOMM34	NA	NA	NA	NA	-0.400	0.252	-0.342	0.212	-0.560	0.082
	NM_006114	TOMM40	NA	NA	NA	NA	0.112	0.854	0.870	0.001	-0.033	1.000
	NM_001286373	TOMM40L	NA	NA	NA	NA	0.155	0.859	0.261	0.461	-0.106	0.962
	NM_001134485	TOMM5	NA	NA	NA	NA	0.027	0.988	0.126	0.710	-0.101	0.931
	NM_001134493	TOMM6	NA	NA	NA	NA	-0.311	0.431	0.340	0.216	-0.158	0.857
	NM_019059	TOMM7	NA	NA	NA	NA	-0.504	0.126	-0.667	0.011	-0.179	0.823
	NM_014820	TOMM70A	NA	NA	NA	NA	0.601	0.051	1.126	0.000	1.051	0.000

G25 + Palm	NM_012456	TIMM10	NA	NA	NA	NA	-0.083	0.902	NA	NA	0.065	0.980
	NM_012192	TIMM10B	NA	NA	NA	NA	-0.073	0.903	NA	NA	0.102	0.924
	NM_012458	TIMM13	NA	NA	NA	NA	-0.193	0.653	NA	NA	-0.140	0.878
	NM_006335	TIMM17A	NA	NA	NA	NA	0.426	0.169	NA	NA	0.263	0.637
	NM_005834	TIMM17B	NA	NA	NA	NA	-0.216	0.603	NA	NA	0.095	0.937
	NM_014177	TIMM21	NA	NA	NA	NA	-0.023	0.983	NA	NA	-0.061	0.980
	NM_013337	TIMM22	NA	NA	NA	NA	-0.067	0.918	NA	NA	0.078	0.962
	NM_006327	TIMM23	NA	NA	NA	NA	0.246	0.547	NA	NA	0.020	1.000
	NM_001290117	TIMM23B	NA	NA	NA	NA	0.197	0.863	NA	NA	0.616	0.529
	NM_006351	TIMM44	NA	NA	NA	NA	0.340	0.374	NA	NA	-0.303	0.641
	NM_001001563	TIMM50	NA	NA	NA	NA	0.038	0.969	NA	NA	0.101	0.936
	NM_004085	TIMM8A	NA	NA	NA	NA	-0.436	0.397	NA	NA	-0.101	0.982
	NM_012459	TIMM8B	NA	NA	NA	NA	0.008	1.000	NA	NA	-0.052	0.984
	NM_001304485	TIMM9	NA	NA	NA	NA	-0.303	0.501	NA	NA	-0.110	0.948
	NM_016589	TIMMDC1	NA	NA	NA	NA	0.062	0.923	NA	NA	0.078	0.956
	NM_014765	TOMM20	NA	NA	NA	NA	0.007	1.000	NA	NA	-0.272	0.644
	NM_020243	TOMM22	NA	NA	NA	NA	-0.062	0.926	NA	NA	-0.060	0.979
	NM_006809	TOMM34	NA	NA	NA	NA	-0.461	0.129	NA	NA	-0.294	0.557
	NM_006114	TOMM40	NA	NA	NA	NA	0.218	0.605	NA	NA	0.397	0.385
	NM_001286373	TOMM40L	NA	NA	NA	NA	0.076	0.946	NA	NA	0.067	0.988
	NM_001134485	TOMM5	NA	NA	NA	NA	0.242	0.521	NA	NA	0.000	1.000
	NM_001134493	TOMM6	NA	NA	NA	NA	-0.081	0.892	NA	NA	-0.090	0.943
	NM_019059	TOMM7	NA	NA	NA	NA	-0.315	0.375	NA	NA	-0.172	0.826
	NM_014820	TOMM70A	NA	NA	NA	NA	0.715	0.010	NA	NA	1.114	0.000

Supplementary Table S5. Quantitative data related to the transcriptomic profiles of mitochondrial iron transport genes in human islets under metabolic stress. NA: Not applicable (condition not tested); ND: not detected.

			Donor #1		Donor #2		Donor #3		Donor #4		Donor #5	
	Accession (NCBI Ref. Sequence)	Gene symbol	**Log₂ FC**	**Adjusted *p***	**Log₂ FC**	**Adjusted *p***	**Log₂ FC**	**Adjusted *p***	**Log₂ FC**	**Adjusted *p***	**Log₂ FC**	**Adjusted *p***
Olea	NM_012089	ABCB10	-0.304	0.493	-0.275	0.648	NA	NA	0.017	1.000	NA	NA
	NM_005689	ABCB6	0.312	0.565	0.321	0.587	NA	NA	0.037	0.971	NA	NA
	NM_007188	ABCB8	1.295	0.002	0.415	0.428	NA	NA	0.651	0.048	NA	NA
	NM_177478	FTMT	ND	ND	ND	ND	NA	NA	ND	ND	NA	NA
	NM_001161706	FXN	-0.901	0.308	-0.131	0.990	NA	NA	0.873	0.198	NA	NA
	NM_031212	SLC25A28	0.286	0.637	0.049	0.976	NA	NA	-0.074	0.921	NA	NA
	NM_016612	SLC25A37	0.618	0.220	0.318	0.630	NA	NA	0.581	0.101	NA	NA
	NM_017875	SLC25A38	-0.693	0.089	0.300	0.568	NA	NA	-0.286	0.473	NA	NA
	NM_016016	SLC25A39	1.051	0.001	0.386	0.359	NA	NA	0.131	0.793	NA	NA
	NM_016462	TMEM14C	-0.323	0.396	0.358	0.430	NA	NA	0.013	1.000	NA	NA
	NM_000714	TSPO	2.680	0.000	0.610	0.163	NA	NA	0.399	0.306	NA	NA
Palm	NM_012089	ABCB10	-0.018	1.000	-0.186	1.000	NA	NA	NA	NA	NA	NA
	NM_005689	ABCB6	0.387	0.473	0.609	1.000	NA	NA	NA	NA	NA	NA
	NM_007188	ABCB8	1.338	0.002	0.502	1.000	NA	NA	NA	NA	NA	NA
	NM_177478	FTMT	ND	ND	ND	ND	NA	NA	NA	NA	NA	NA
	NM_001161706	FXN	-2.152	0.022	-0.943	1.000	NA	NA	NA	NA	NA	NA
	NM_031212	SLC25A28	0.653	0.173	-0.101	1.000	NA	NA	NA	NA	NA	NA
	NM_016612	SLC25A37	-0.113	0.953	0.017	1.000	NA	NA	NA	NA	NA	NA
	NM_017875	SLC25A38	-0.104	0.901	0.105	1.000	NA	NA	NA	NA	NA	NA
	NM_016016	SLC25A39	1.056	0.001	0.142	1.000	NA	NA	NA	NA	NA	NA
	NM_016462	TMEM14C	0.061	0.938	0.300	1.000	NA	NA	NA	NA	NA	NA
	NM_000714	TSPO	2.058	0.000	0.104	1.000	NA	NA	NA	NA	NA	NA

G25	NM_012089	ABCB10	-0.311	0.440	-0.856	0.068	-0.528	0.319	-0.400	0.199	NA	NA
	NM_005689	ABCB6	0.007	1.000	0.279	0.623	-0.145	0.819	0.004	1.000	NA	NA
	NM_007188	ABCB8	1.218	0.004	0.569	0.202	-0.209	0.708	0.878	0.001	NA	NA
	NM_177478	FTMT	ND	ND	ND	ND	ND	ND	ND	ND	NA	NA
	NM_001161706	FXN	-1.268	0.162	-0.273	0.884	-0.113	0.919	0.618	0.354	NA	NA
	NM_031212	SLC25A28	0.430	0.387	0.426	0.348	0.380	0.770	0.801	0.003	NA	NA
	NM_016612	SLC25A37	0.498	0.297	-0.286	0.692	0.355	0.352	0.156	0.692	NA	NA
	NM_017875	SLC25A38	-0.428	0.287	0.221	0.673	-0.449	0.254	0.188	0.562	NA	NA
	NM_016016	SLC25A39	1.312	0.000	0.486	0.152	0.263	0.538	0.607	0.018	NA	NA
	NM_016462	TMEM14C	-0.215	0.558	0.328	0.416	-0.051	0.964	0.083	0.827	NA	NA
	NM_000714	TSPO	3.122	0.000	0.894	0.014	0.401	0.278	-0.486	0.123	NA	NA
G25+Olea+Palm	NM_012089	ABCB10	NA	NA	NA	NA	-0.236	0.787	NA	NA	0.609	0.342
	NM_005689	ABCB6	NA	NA	NA	NA	-0.111	0.877	NA	NA	-0.129	0.973
	NM_007188	ABCB8	NA	NA	NA	NA	-0.060	0.951	NA	NA	0.372	0.574
	NM_177478	FTMT	NA	NA	NA	NA	ND	ND	NA	NA	ND	ND
	NM_001161706	FXN	NA	NA	NA	NA	-0.414	0.514	NA	NA	-0.366	0.754
	NM_031212	SLC25A28	NA	NA	NA	NA	0.571	0.576	NA	NA	-0.278	0.979
	NM_016612	SLC25A37	NA	NA	NA	NA	0.030	0.983	NA	NA	-0.211	0.899
	NM_017875	SLC25A38	NA	NA	NA	NA	-0.349	0.433	NA	NA	-0.279	0.756
	NM_016016	SLC25A39	NA	NA	NA	NA	0.336	0.385	NA	NA	0.155	0.928
	NM_016462	TMEM14C	NA	NA	NA	NA	0.121	0.849	NA	NA	0.015	1.000
	NM_000714	TSPO	NA	NA	NA	NA	0.477	0.161	NA	NA	0.220	0.868
G25+Olea	NM_012089	ABCB10	NA	NA	NA	NA	-0.124	0.906	-0.090	0.810	0.696	0.158
	NM_005689	ABCB6	NA	NA	NA	NA	-0.284	0.521	0.106	0.757	-0.550	0.159
	NM_007188	ABCB8	NA	NA	NA	NA	-0.036	0.982	1.272	0.000	0.044	0.996
	NM_177478	FTMT	NA	NA	NA	NA	ND	ND	ND	ND	ND	ND
	NM_001161706	FXN	NA	NA	NA	NA	-0.244	0.732	0.547	0.396	-1.110	0.010
	NM_031212	SLC25A28	NA	NA	NA	NA	0.671	0.360	1.037	0.000	0.313	0.857

	NM_016612	SLC25A37	NA	NA	NA	NA	0.146	0.785	0.322	0.298	-0.229	0.804
	NM_017875	SLC25A38	NA	NA	NA	NA	-0.612	0.066	-0.017	0.975	-0.683	0.048
	NM_016016	SLC25A39	NA	NA	NA	NA	0.359	0.312	0.715	0.003	-0.070	0.961
	NM_016462	TMEM14C	NA	NA	NA	NA	-0.036	0.977	-0.089	0.794	0.088	0.946
	NM_000714	TSPO	NA	NA	NA	NA	0.634	0.030	-0.066	0.878	0.403	0.389
G25+Palm	NM_012089	ABCB10	NA	NA	NA	NA	0.379	0.423	NA	NA	0.655	0.212
	NM_005689	ABCB6	NA	NA	NA	NA	-0.108	0.852	NA	NA	-0.292	0.649
	NM_007188	ABCB8	NA	NA	NA	NA	-0.507	0.128	NA	NA	0.350	0.522
	NM_177478	FTMT	NA	NA	NA	NA	ND	ND	NA	NA	ND	ND
	NM_001161706	FXN	NA	NA	NA	NA	0.141	0.848	NA	NA	-0.287	0.777
	NM_031212	SLC25A28	NA	NA	NA	NA	0.792	0.194	NA	NA	0.436	0.744
	NM_016612	SLC25A37	NA	NA	NA	NA	1.000	0.000	NA	NA	-0.190	0.851
	NM_017875	SLC25A38	NA	NA	NA	NA	-0.642	0.035	NA	NA	-0.178	0.843
	NM_016016	SLC25A39	NA	NA	NA	NA	0.258	0.479	NA	NA	0.246	0.673
	NM_016462	TMEM14C	NA	NA	NA	NA	0.128	0.801	NA	NA	0.004	1.000
	NM_000714	TSPO	NA	NA	NA	NA	0.677	0.013	NA	NA	0.258	0.704

Supplementary Table S6. Quantitative data related to the transcriptomic profiles of mitochondrial calcium transport genes in human islets upon metabolic stress. NA: Not applicable (condition not tested); ND: not detected.

			Donor #1		Donor #2		Donor #3		Donor #4		Donor #5	
	Accession (NCBI Ref. Sequence)	Gene symbol	**Log2 FC**	**Adjusted *p***	**Log2 FC**	**Adjusted *p***	**Log2 FC**	**Adjusted *p***	**Log2 FC**	**Adjusted *p***	**Log2 FC**	**Adjusted *p***
Olea	NM_001270679	MCU	-0.045	0.974	-0.080	0.933	NA	NA	-0.134	0.801	NA	NA
	NM_001031713	MCUR1	-0.081	0.913	-0.131	0.841	NA	NA	0.116	0.833	NA	NA
	NM_006077	MICU1	-0.282	0.503	0.094	0.895	NA	NA	-0.354	0.331	NA	NA
	NM_152726	MICU2	-0.852	0.005	0.134	0.832	NA	NA	-0.154	0.768	NA	NA
	NM_181723	MICU3	-0.939	0.008	-0.526	0.400	NA	NA	0.183	0.746	NA	NA
	NM_024959	SLC8B1	0.742	0.104	0.546	0.309	NA	NA	-0.113	0.871	NA	NA
	NM_033318	SMDT1	0.754	0.048	0.289	0.558	NA	NA	0.101	0.857	NA	NA
Palm	NM_001270679	MCU	-0.032	0.997	-0.030	1.000	NA	NA	NA	NA	NA	NA
	NM_001031713	MCUR1	-0.188	0.767	-0.048	1.000	NA	NA	NA	NA	NA	NA
	NM_006077	MICU1	0.075	0.935	-0.146	1.000	NA	NA	NA	NA	NA	NA
	NM_152726	MICU2	-0.300	0.450	0.107	1.000	NA	NA	NA	NA	NA	NA
	NM_181723	MICU3	-0.562	0.161	0.289	1.000	NA	NA	NA	NA	NA	NA
	NM_024959	SLC8B1	0.276	0.719	-0.676	1.000	NA	NA	NA	NA	NA	NA
	NM_033318	SMDT1	0.918	0.013	0.260	1.000	NA	NA	NA	NA	NA	NA
G25	NM_001270679	MCU	0.109	0.828	-0.075	0.947	0.040	0.980	-0.686	0.012	NA	NA
	NM_001031713	MCUR1	0.073	0.934	-0.407	0.348	0.000	1.000	0.231	0.459	NA	NA
	NM_006077	MICU1	-0.100	0.847	-0.092	0.891	-0.078	0.924	0.635	0.014	NA	NA
	NM_152726	MICU2	-0.819	0.006	0.273	0.545	-0.304	0.476	0.269	0.395	NA	NA
	NM_181723	MICU3	-1.102	0.002	-0.640	0.349	-0.200	0.810	-0.106	0.820	NA	NA
	NM_024959	SLC8B1	0.913	0.025	0.394	0.498	0.766	0.013	-0.593	0.082	NA	NA
	NM_033318	SMDT1	1.093	0.001	0.789	0.018	-0.472	0.155	-0.048	0.910	NA	NA
G25+ Olea+ Palm	NM_001270679	MCU	NA	NA	NA	NA	0.009	1.000	NA	NA	-0.323	0.650
	NM_001031713	MCUR1	NA	NA	NA	NA	0.198	0.731	NA	NA	0.257	0.807

	NM_006077	MICU1	NA	NA	NA	NA	-0.047	0.963	NA	NA	0.061	1.000
	NM_152726	MICU2	NA	NA	NA	NA	-0.091	0.902	NA	NA	-0.226	0.821
	NM_181723	MICU3	NA	NA	NA	NA	-0.779	0.123	NA	NA	-0.343	0.742
	NM_024959	SLC8B1	NA	NA	NA	NA	0.647	0.053	NA	NA	0.216	0.923
	NM_033318	SMDT1	NA	NA	NA	NA	-0.435	0.205	NA	NA	-0.063	1.000
G25+Olea	NM_001270679	MCU	NA	NA	NA	NA	0.014	1.000	-0.239	0.426	0.081	0.962
	NM_001031713	MCUR1	NA	NA	NA	NA	-0.028	0.990	-0.034	0.934	0.793	0.011
	NM_006077	MICU1	NA	NA	NA	NA	-0.116	0.845	0.328	0.230	0.165	0.838
	NM_152726	MICU2	NA	NA	NA	NA	-0.381	0.301	0.302	0.295	-0.087	0.949
	NM_181723	MICU3	NA	NA	NA	NA	-0.786	0.081	0.090	0.822	0.230	0.804
	NM_024959	SLC8B1	NA	NA	NA	NA	0.819	0.006	-0.453	0.177	0.071	0.988
	NM_033318	SMDT1	NA	NA	NA	NA	-0.410	0.224	0.077	0.823	0.071	0.965
G25+Palm		MCU	NA	NA	NA	NA	0.136	0.793	NA	NA	0.081	0.962
		MCUR1	NA	NA	NA	NA	0.161	0.749	NA	NA	0.793	0.011
		MICU1	NA	NA	NA	NA	-0.012	1.000	NA	NA	0.165	0.838
		MICU2	NA	NA	NA	NA	-0.169	0.711	NA	NA	-0.087	0.949
		MICU3	NA	NA	NA	NA	-0.143	0.855	NA	NA	0.230	0.804
		SLC8B1	NA	NA	NA	NA	0.656	0.030	NA	NA	0.071	0.988
		SMDT1	NA	NA	NA	NA	-0.458	0.124	NA	NA	0.071	0.965

Species	Accession NCBI Ref Seq.	Primer name	Sequence
Homo sapiens	NM_001358345.2	Solute carrier family 25 member 10 (SLC25A10), transcript variant 1, mRNA	Fwd: 5’- ATCCTGGCACTCTACAGCGG -3’<br>Rev: 5’- GTCTCGTAGATGGCGAACCG -3’
Homo sapiens	NM_003562.5	Solute carrier family 25 member 11 (SLC25A11), transcript variant 1, mRNA	Fwd: 5’-TGCCTTTGTGGGAACACCAG-3’<br>Rev: 5’-ACATTTTTGTAGCCACGGCG-3’
Homo sapiens	NM_001191060.2	Solute carrier family 25 member 22 (SLC25A22), transcript variant 1, mRNA	Fwd: 5’- GTGGCATTGCCGGTCTCTAC-3’<br>Rev: 5’- GAGCGGGAAGTACACCACAG-3’
Homo sapiens	NM_001358345.2	Solute carrier family 8 member B1 (SLC8B1), transcript variant 3, mRNA	Fwd: 5’- CCAGCTTCAGGTGTGAACCAG-3’<br>Rev:5’- GAAGTCACAGCGGTCAGAGACA-3’
Homo sapiens	NM_012458.4	Translocase of inner mitochondrial membrane 13 (TIMM13), mRNA	Fwd: 5’- GTGAAAGTGCAGATCGCCGT-3’<br>Rev:5’- AGCCCCCAGGTTTCCCTATAC-3’
Homo sapiens	NM_021130.5	Peptidylprolyl isomerase A (PPIA), transcript variant 1, mRNA	Fwd: 5’- ATCTGCACTGCCAAGACTGA-3’<br>Rev: 5’-TCTTGCTGGTCTTGCCATTC-3’

Supplementary Table S7: Primers used for quantitative RT-PCR analysis

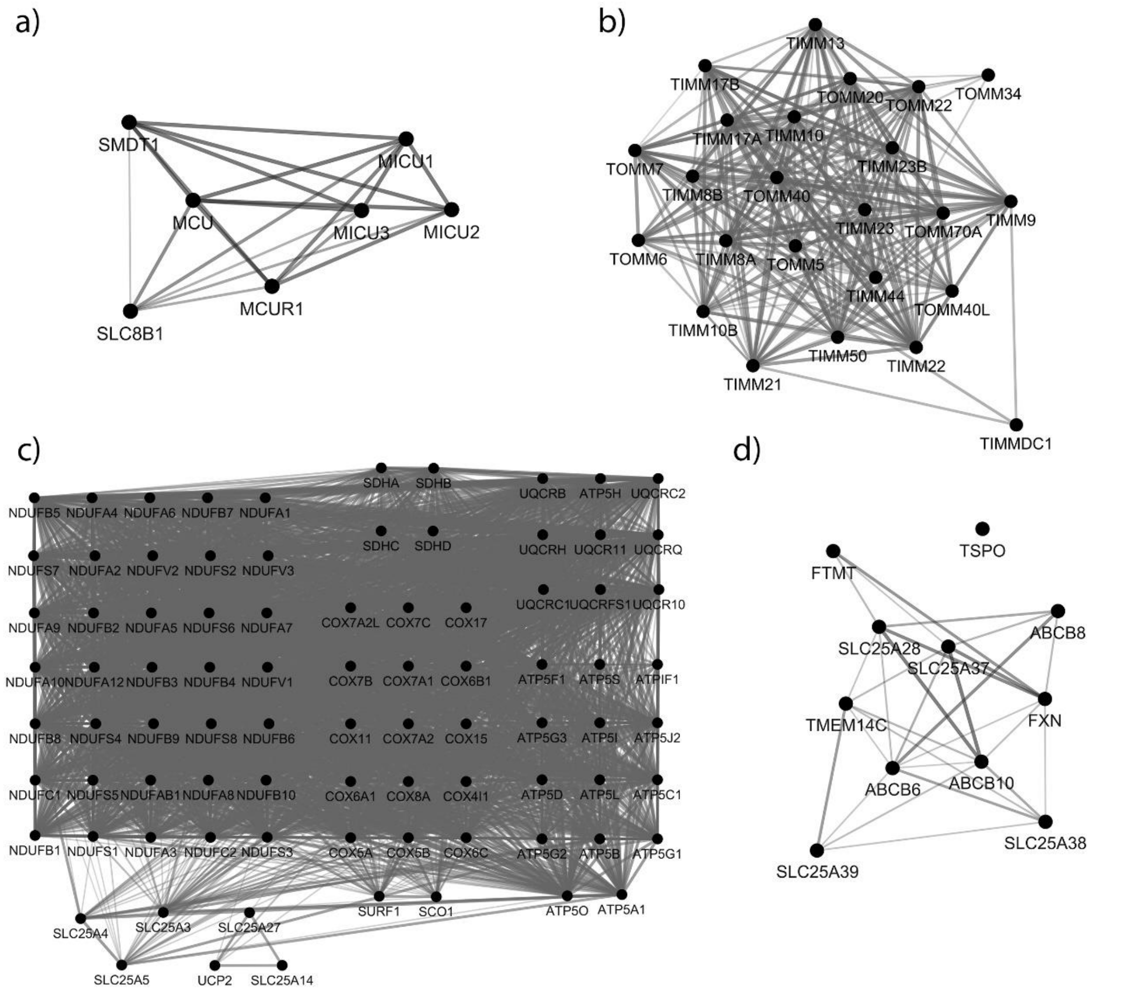

Supplementary Figure S1. Functional interaction network of human (**a**) mitochondrial calcium transport genes; (**b**) outer and inner mitochondrial membrane translocases TOM/TIM machinery; (**c**) electron transport chain machinery and related carriers; (**d**) mitochondrial iron transport genes. Nodes were connected using the STRING interaction knowledgebase with a confidence score >0.4.

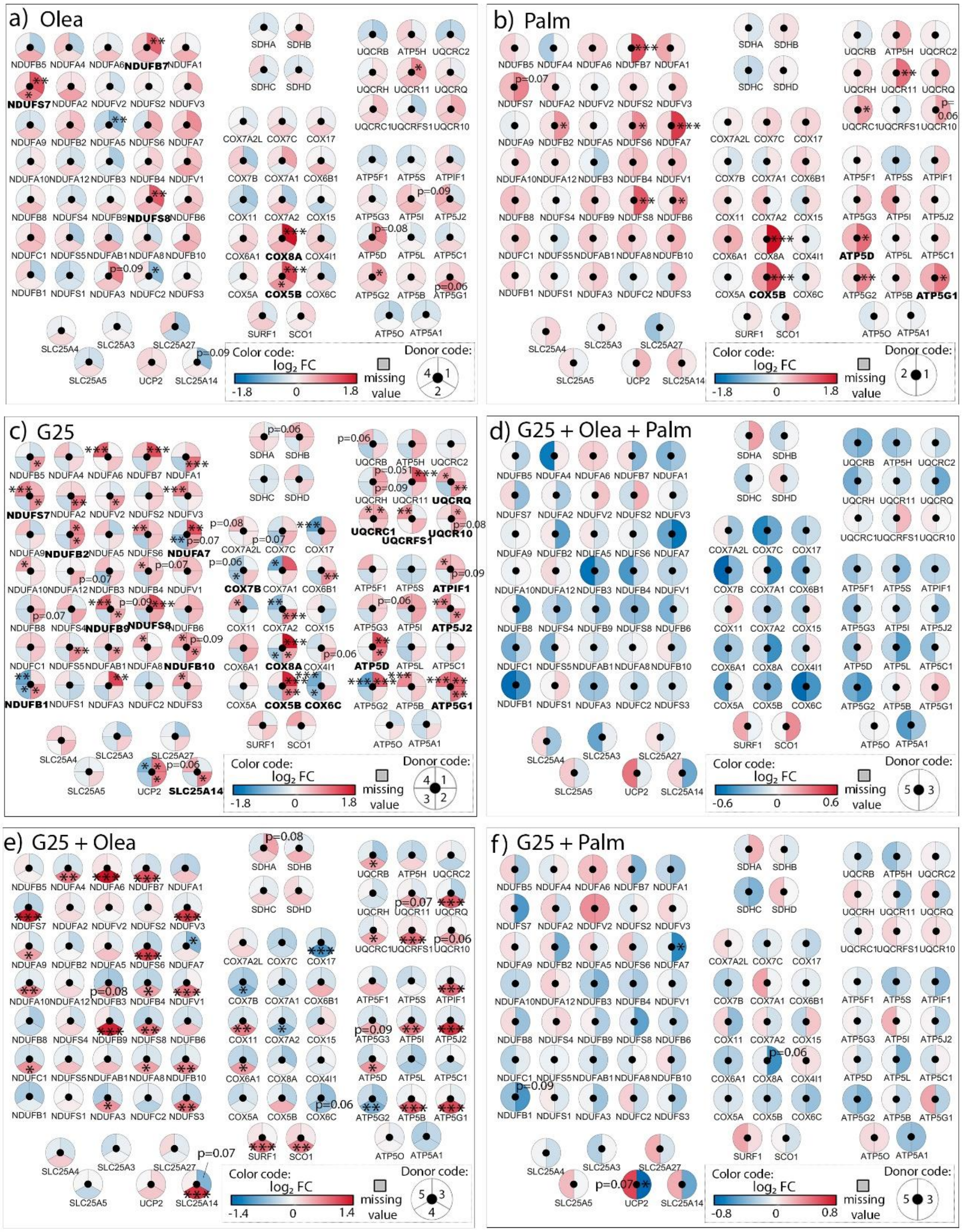

Supplementary Figure S2. Effects of high 25 mM glucose (G25) and 0.4 mM oleate (Olea) or palmitate (Palm) on the transcriptional regulation of the electron transport chain machinery. Human islets were exposed to (**a**) Olea at G5.5, (**b**) Palm at G5.5, (**c**) G25, (**d**) G25+Olea+Palm, (**e**) G25+Olea, and (**f**) G25+Palm for 3 days before RNA-Seq analysis. Effects of culture conditions on transcript levels are compared to standard G5.5 medium and shown as upregulated (red), downregulated (blue), or unchanged (white). Missing values are represented in grey. Each disk is split into individual changes for the different donors. Color code reflects the transcriptional changes in log_2 fold changes (log_2 FC) for that particular gene in individual donors. *adjusted $p<0.05$, **adjusted $p<0.01$, ***adjusted $p<0.001$ between control 5.5 mM glucose and the specific culture condition.

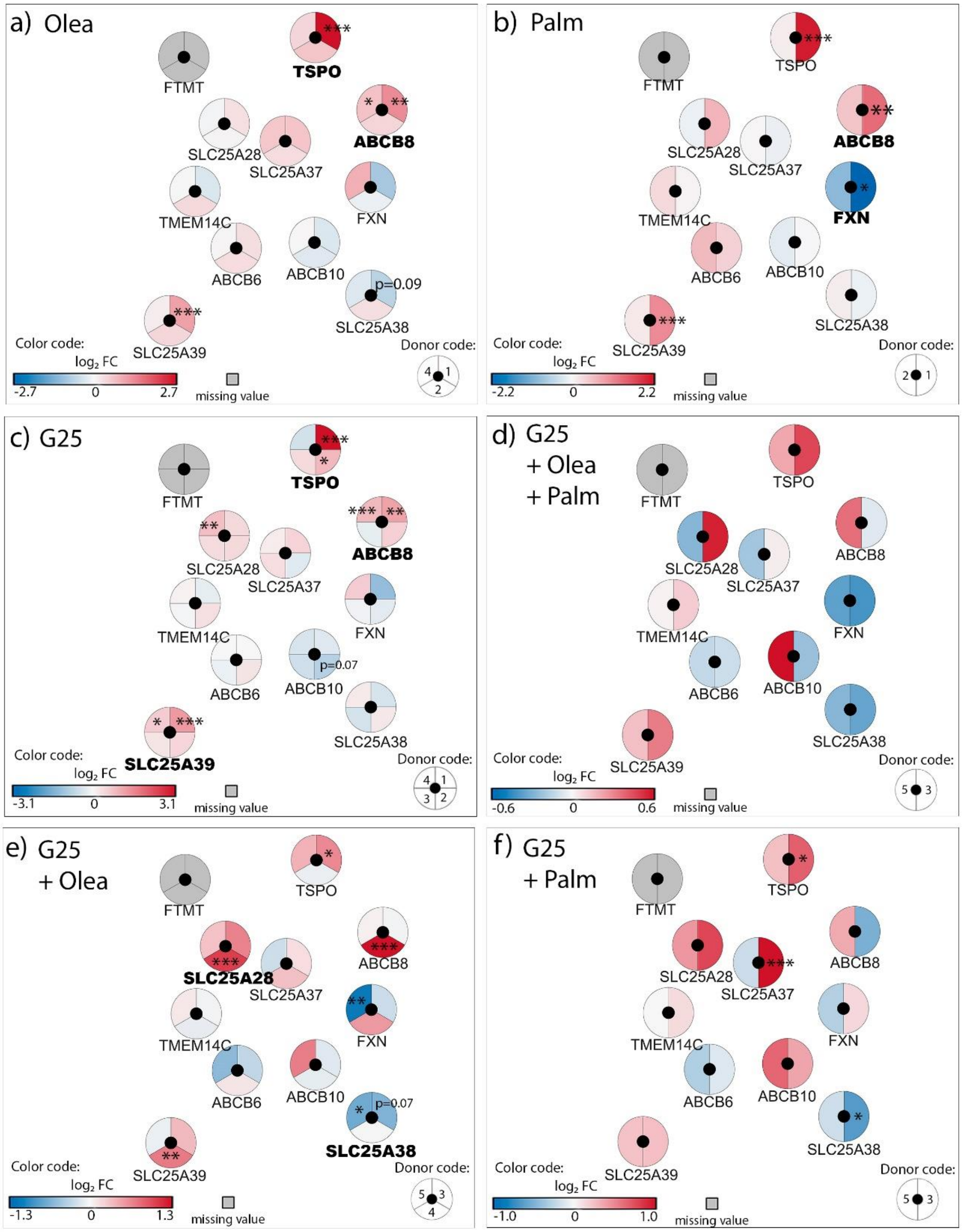

Supplementary Figure S3. Effects of high 25 mM glucose (G25) and 0.4 mM oleate (Olea) or palmitate (Palm) on the transcriptional regulation of mitochondrial iron transport genes. Human islets were exposed to (**a**) Olea at G5.5, (**b**) Palm at G5.5, (**c**) G25, (**d**) G25+Olea+Palm, (**e**) G25+Olea, and (**f**) G25+Palm for 3 days before RNA-Seq analysis. Effects of culture conditions on transcript levels are compared to standard G5.5 medium and shown as upregulated (red), downregulated (blue), or unchanged (white). Missing values are represented in grey. Each disk is split into individual changes for the different donors. Color code reflects the transcriptional changes in $\log_2$ fold changes ($\log_2$ FC) for that particular gene in individual donors. *adjusted $p<0.05$, **adjusted $p<0.01$, ***adjusted $p<0.001$ between control 5.5 mM glucose and the specific culture condition.

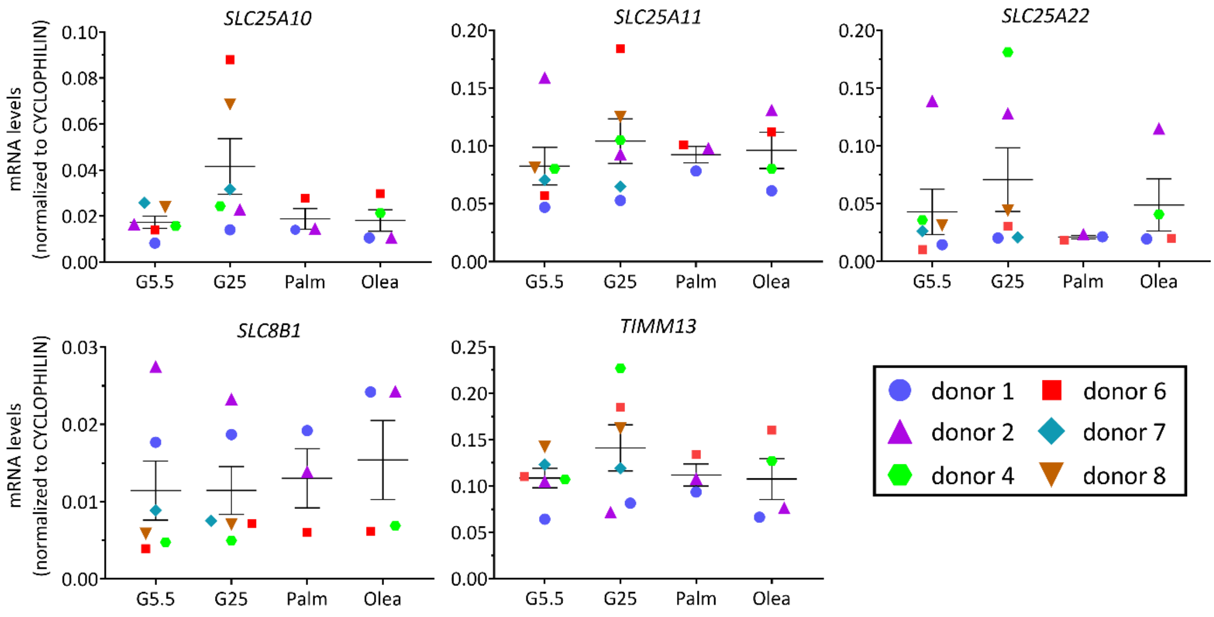

Supplementary Figure S4: Effects of high 25 mM glucose (G25) and 0.4 mM palmitate (Palm) or oleate (Olea) on mRNA levels of selected genes in human islets measured by quantitative RT–PCR, normalized to cyclophilin A (*PPIA*). See Supplementary Table S1 for details on the donors.
